# Functional Trade-Offs in Promiscuous Enzymes Cannot Be Explained by Intrinsic Mutational Robustness of the Native Activity

**DOI:** 10.1371/journal.pgen.1006305

**Published:** 2016-10-07

**Authors:** Miriam Kaltenbach, Stephane Emond, Florian Hollfelder, Nobuhiko Tokuriki

**Affiliations:** 1 Michael Smith Laboratories, University of British Columbia, Vancouver, Canada; 2 Department of Biochemistry, University of Cambridge, Cambridge, United Kingdom; University of Michigan, UNITED STATES

## Abstract

The extent to which an emerging new function trades off with the original function is a key characteristic of the dynamics of enzyme evolution. Various cases of laboratory evolution have unveiled a characteristic trend; a large increase in a new, promiscuous activity is often accompanied by only a mild reduction of the native, original activity. A model that associates weak trade-offs with “evolvability” was put forward, which proposed that enzymes possess mutational robustness in the native activity and plasticity in promiscuous activities. This would enable the acquisition of a new function without compromising the original one, reducing the benefit of early gene duplication and therefore the selection pressure thereon. Yet, to date, no experimental study has examined this hypothesis directly. Here, we investigate the causes of weak trade-offs by systematically characterizing adaptive mutations that occurred in two cases of evolutionary transitions in enzyme function: (1) from phosphotriesterase to arylesterase, and (2) from atrazine chlorohydrolase to melamine deaminase. Mutational analyses in various genetic backgrounds revealed that, in contrast to the prevailing model, the native activity is less robust to mutations than the promiscuous activity. For example, in phosphotriesterase, the deleterious effect of individual mutations on the native phosphotriesterase activity is much larger than their positive effect on the promiscuous arylesterase activity. Our observations suggest a revision of the established model: weak trade-offs are not caused by an intrinsic robustness of the native activity and plasticity of the promiscuous activity. We propose that upon strong adaptive pressure for the new activity without selection against the original one, selected mutations will lead to the largest possible increases in the new function, but whether and to what extent they decrease the old function is irrelevant, creating a bias towards initially weak trade-offs and the emergence of generalist enzymes.

## Author Summary

Understanding how enzymes evolve is a fundamental question that can help us decipher not only the mechanisms of evolution on a higher level, *i*.*e*., whole organisms, but also advances our knowledge of sequence-structure-function relationships as a guide to artificial evolution in the test tube. An important yet unexplained phenomenon occurs during the evolution of a new enzymatic function; it has been observed that new and ancestral functions often trade-off only weakly, meaning the original native activity is initially maintained at a high level despite drastic improvement of the new promiscuous activity. It has previously been proposed that weak trade-offs occur because the native activity is robust to mutations while the promiscuous activity is not. However, the present work contradicts this hypothesis, based on the detailed characterization of mutational effects on both activities in two examples of enzyme evolution. We propose an alternative explanation: the weak activity trade-off is consistent with being a by-product of strong selection for the new activity rather than an intrinsic property of the native activity.

## Introduction

**Fig 1:**
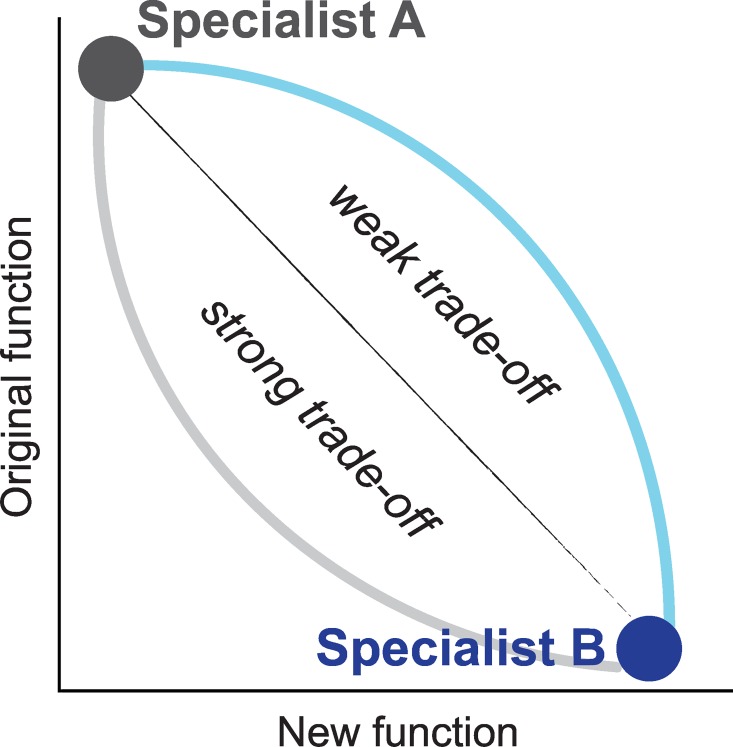
Functional trade-offs in protein evolution. Strong trade-offs result when mutations increasing the new function have a large effect on the original function. When the effect on the original function is mild, trade-offs are weak. Weak trade-offs channel evolution through a generalist regime where the enzyme catalyzes both reactions with high efficiency.

The evolution of new enzymatic functions commonly occurs via the modification of existing enzymes that exhibit a promiscuous activity, increasing this activity through adaptive mutations, and eventually duplicating the encoding gene to generate a new enzyme [1–6]. This process has driven the emergence of a large repertoire of functions in enzyme superfamilies [7–12]. As many modern enzymes are highly specialized for a single chemical reaction, and exhibit several orders of magnitude lower rates for promiscuous activities [13–16], it is assumed that adaptation towards a new function involves a trade-off with the original function [4]. The extent of this trade-off determines how long an enzyme can maintain catalysis of both chemical reactions as a bifunctional, generalist enzyme, and the point at which gene duplication becomes essential to diverge into two new specialists [6, 17]. If trade-offs are strong and the gain of new function comes at a significant cost to the original one (**Fig 1**), gene duplication at an early stage of the adaptive process is indispensable, otherwise adaptation will be constrained as long as the original function remains necessary. On the other hand, if trade-offs are weak and the new function can develop while a high level of the original one is maintained (**Fig 1**), the timing of gene duplication is less crucial.

Growing evidence from experimental evolution has revealed a strong empirical trend; laboratory evolution experiments that select for significant increases in a promiscuous activity (100-1000-fold) have typically resulted in only a marginal decrease (~10-fold) in the original activity [3, 4, 18]. Thus, trade-offs appear to be weak at the early stages of adaptation, and functional transitions tend to proceed via generalist intermediates [4, 18–31]. However, what causes the weak trade-offs observed in these studies remains unclear. To date, only one mechanistic model has been proposed: Tawfik and co-workers suggested that weak trade-offs result because enzymes are inherently evolvable: they are able to promptly respond to a selection pressure toward a new function without compromising their native activity. In other words, enzymes possess “robustness” (tolerance or insensitivity) to mutational perturbations in their native activity, but are endowed with “plasticity” (malleability or sensitivity) in their promiscuous activities (dubbed the “robustness model” in this work) [4, 18, 32]. However, to date no experimental study examining this hypothesis is on record. Our present work raises the possibility that the observations from laboratory evolution may be biased because the selection pressure to increase the new function is typically high in these experiments, and there is no selection pressure against the original activity. As a result, laboratory evolutionary trajectories follow highly adaptive pathways and only mutations that strongly increase the target activity are sampled and isolated. Therefore, conclusions drawn from these observations may be biased and a more comprehensive analysis is required in order to evaluate the model.

In this work, we examine the robustness model by comprehensively analyzing the mutations that collectively cause a complete functional switch from original to new function within two examples of evolution. We characterize the effect of each mutation on both functions and do so in various mutational backgrounds: in the wild-type enzyme (“the evolutionary starting point”), the evolved enzyme (“the new specialist enzyme”) and at the time of occurrence during the evolutionary trajectory. Our first example is a phosphotriesterase (PTE), which was evolved into an efficient arylesterase (AE) using laboratory evolution [29, 33], and the other is the natural evolutionary transition from atrazine chlorohydrolase (AztA) to deaminase (TriA) [34]. We also characterize > 400 random mutations in wild-type PTE to extend our analysis beyond adaptive mutations and gain more general insights. Our results suggest that the observed weak trade-offs are not caused by the mutational robustness of the native activity and plasticity of the new activity and thus, the robustness model does not apply. Instead, we propose that weak trade-offs are the by-product of strong selection pressure for the new function.

## Results

### The weak activity trade-off in the evolution of PTE is not caused by robustness to mutational perturbations

The robustness model proposed by Tawfik and co-workers argues that weak trade-offs are observed because enzymes possess mutational robustness in their native activity, and plasticity in promiscuous activities. In order to test this hypothesis, we comprehensively quantified the effect of individual mutations on two specialized enzymes, *i*.*e*., on an evolutionary starting and end point. If the model is true, the mutational effect on the native activity should be relatively marginal whereas the effect on the promiscuous activity should be larger on average.

**Fig 2:**
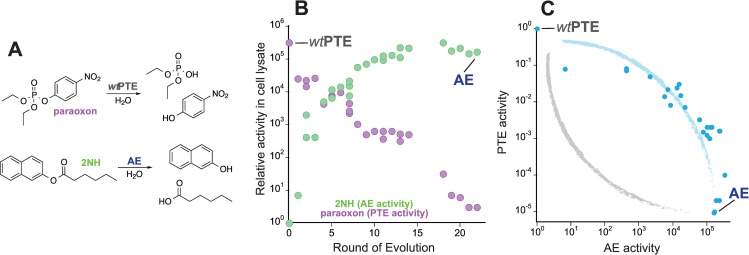
Evolution from *wt*PTE to AE. (A) *wt*PTE catalyzes the hydrolysis of paraoxon (PTE activity). AE catalyzes 2-naphthyl hexanoate (2NH) hydrolysis (AE activity). The two specialist enzymes catalyze each other’s reaction promiscuously. (B) Development of PTE and AE activities during the directed evolution experiment. (C) The trade-off between the two activities over the evolution is weak. To illustrate, the idealized weak (blue) and strong (grey) trade-off curves shown in Fig 1 are indicated. Each variant is shown as a blue dot.

We used a bacterial phosphotriesterase (PTE) as a model system. Our previous directed evolution experiment from wild-type phosphotriesterase (*wt*PTE) towards arylesterase (AE) followed a weak trade-off trajectory through the accumulation of 26 mutations over 22 rounds of directed evolution (**Fig 2A and 2B, S1 Table**) [29, 33, 35, 36]. Trade-offs between the two catalytic activities were initially weak (i.e., loosing one order of magnitude in the native activity but gaining 10^4^ in the promiscuous activity until round 6), and became stronger in the later rounds (now losing 10^4^-fold while only gaining another 10-fold). Thus, the transition between the original and new activity follows a concave curve, which is characteristic for weak trade-offs during the early stages of evolution described in **Fig 1**(see also **Fig 2C)**. Overall, the 26 mutations resulted in the same magnitude of change in the two activities: a 10^5^-fold increase in the new arylesterase activity (10^4^-fold in terms of *k*_*cat*_/*K*_*M*_), and a 10^5^-fold decrease in the original phosphotriesterase activity (*k*_*cat*_/*K*_*M*_: 10^4^-fold). Examining the individual effect of each mutation on the starting and end point of the evolution enables us to determine whether the weak trade-off observed during the evolution is supported by robustness of the native activity.

**Fig 3:**
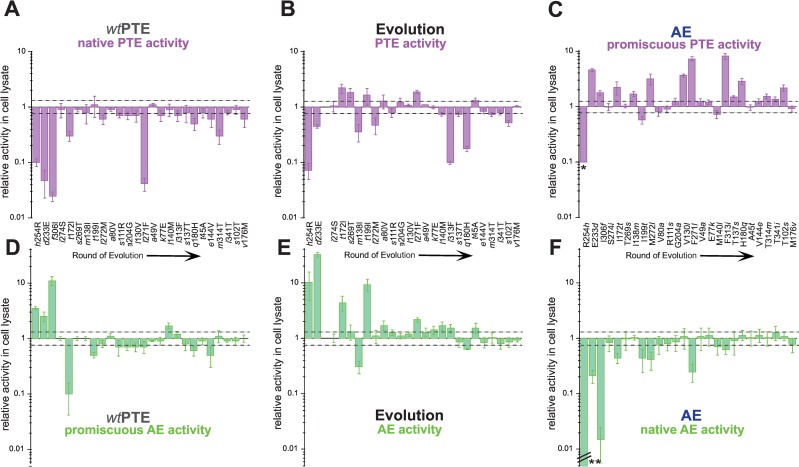
Effect of all single point mutations obtained over the evolution. (A)-(C) Effect of mutations on PTE activity (A) in the *wt*PTE background, (B) upon their occurence in the evolution, and (C) in the AE background. *Phosphotriesterase activity was too low to be determined in AE-R254*h*, but at least 10-fold reduced compared to AE. (D)-(F) Effect of mutations on AE activity (D) in the *wt*PTE background, (E) upon their occurrence in the evolution, and (F) in the AE background. **Arylesterase activity was reduced to 1.9×10^−5^ times the level of AE and is therefore not shown to scale. Activities are given relative to the respective parent background. Mutations causing a >1.3-fold change compared to the parent mutant (dotted line) are considered non-neutral. A student T-test was performed and p-values compared to each parent were calculated (**S2–S4 Tables**). The 1.3-fold effect of T341*i* on AE activity in the AE background as well as the effect of *t*199I, *l*140M and *t*45A on PTE activity in the evolution is statistically not significant. Note that in the evolution, *f*306 was first mutated to L and then to I and therefore, the direct effect of *f*306I could not be determined. Amino acids found in *wt*PTE are shown in lower-case italics.

The robustness model predicts that individual mutations in the background of *wt*PTE should have a small effect on the phosphotriesterase activity and a large effect on the arylesterase activity. To assess whether or not this is true, we generated 26 single point mutants of *wt*PTE (introducing each of the mutations that accumulated over the evolution) and AE (reverting each of the mutations back to the amino acid found in *wt*PTE) as well as selected intermediate variants, and analyzed the effect of individual mutations upon three genetic backgrounds: (i) the evolutionary starting point, *wt*PTE, (ii) the endpoint, AE, and (iii) the point of occurrence in the evolutionary trajectory [29]. We then assayed all mutants for total phosphotriesterase and arylesterase activity in clarified cell lysate (**Fig 3, S2–S4 Tables**). Total activity is a combination of intrinsic enzymatic activity and soluble expression levels, which is the appropriate measure for comparison since it reflects “variant fitness” in our evolutionary model system. We have previously shown that total and intrinsic activities are well correlated [27], partly because all variants were co-expressed with the chaperones GroEL/ES, minimizing fluctuations in soluble expression levels. Moreover, fluctuations in soluble expression will affect both activities to the same extent and therefore all conclusions about trade-offs are independent of expression effects. Contrary to the expectations of the robustness model, which states that the original activity is robust in the initial (wild-type) background, we observed that mutations are significantly more deleterious for the native, phosphotriesterase activity in the *wt*PTE background when compared to their effects in the evolutionary trajectory (**Fig 3A–3C)**. Assuming perfect additivity and no epistasis (*i*.*e*., the null-model), a 10^9^-fold reduction in phosphotriesterase activity is expected when all mutations are combined (calculated by the sum of effects from all mutations; corresponding to an average 0.46-fold reduction of the wild type activity level per mutation, **Table 1**). This calculated value far exceeds the actual 10^5^-fold change observed in the trajectory (average 0.71-fold reduction per mutation, **Table 1**). Second, in arylesterase activity, the overall effect of individual mutations in *wt*PTE is significantly weaker: the predicted change in arylesterase according to the null-model is a 2-fold decrease (average mutational change of 0.98, **Table 1**), which is considerably less than the 10^5^–fold increase observed (average 1.57-fold mutational effect, **Fig 3D–3F**, **Table 1**). Thus, the mutational effects on phosphotriesterase activity are far larger than their effects on arylesterase activity in the background of *wt*PTE (null model: 10^9^-fold vs 10-fold), indicating that *wt*PTE reacts much stronger to mutations in its native phosphotriesterase activity compared to its promiscuous arylesterase activity.

**Table 1:** Distribution of mutational effects in the evolution of PTE and AtzA.

**PTE evolution**	***wt*PTE**		**Evolution**		**AE**	
	**paraoxon**	**2NH**	**paraoxon**	**2NH**	**paraoxon**	**2NH**
*Deleterious mutations*^*[*^^*a*^^*]*^	15	8	10	2	3	8
*Neutral mutations*^*[*^^*a*^^*]*^	11	14	10	10	10	18
*Favorable mutations*^*[*^^*a*^^*]*^	0	4	5	13	13	0
*Average mutational change*^*[*^^*b*^^,^ ^*c*^^*]*^	0.46 (0.31;0.70)	0.98 (0.73;1.31)	0.71 (0.52;1.01)	1.57 (1.07;2,30)	1.47 (1.05;2.06)	0.43 (0.18;1.02)
*Median mutational change*^*[*^^*b*^^*]*^	0.71	0.91	0.84	1.27	1.31	0.85
*Expected total change*^*[*^^*b*^^,^ ^*d*^^*]*^	1.8×10^−9^	5.8×10^−1^	3.2×10^−4^	7.3×10^4^	2.4×10^4^	3.7×10^−10^
*Observed total change*^*[*^^*b*^^,^ ^*e*^^*]*^	/	/	(1.0±0.01)×10^−5^	(1.7±0.6)×10^5^	/	/
**AtzA evolution**	**AtzA**		**Evolution**		**TriA**	
	**atrazine**	**melamine**	**atrazine**	**melamine**	**atrazine**	**melamine**
*Deleterious mutations*^*[*^^*a*^^*]*^	3	0	5	0	1	7
*Neutral mutations*^*[*^^*a*^^*]*^	6	8	4	4	4	2
*Favorable mutations*^*[*^^*a*^^*]*^	0	1	0	5	4	0
*Average mutational change*^*[*^^*b*^^,^ ^*c*^^*]*^	0.5 (0.23;1.06)	/	0.54 (0.35;0.82)	/	/	/
*Median mutational change*^*[*^^*b*^^*]*^	1	/	0.7	/	/	/
*Expected total change*^*[*^^*b*^^,^ ^*d*^^*]*^	1.8×10^−3^	/	4.1×10^−3^	/	/	/
*Observed total change*^*[*^^*b*^^,^ ^*e*^^*]*^	/	/	2.4×10^−2^	/	/	/

[a] Mutations are considered deleterious if they cause a >1.3-fold reduction in activity compared to the respective parent, favorable if they cause a >1.3-fold increase and otherwise neutral. A student t-test was performed to obtain p-values (S2–S4 Tables). Only mutants with an average >1.3-fold change AND a p-value <0.05 are considered significant.

Note that in the PTE evolution, *f*306 was first mutated to L and then to I and the direct effect of *f*306I could not be calculated. Therefore, the number of mutations adds up to only 25 instead of 26.

[b] Values are given relative to the respective parent variant. Several numbers could not be calculated because at least one variant showed no detectable activity.

[c] The average mutational change was calculated as the geometric mean of the relative activities of all variants (see Figs 3 and 6) and the 95% confidence interval is indicated between brackets.

[d] The expected total change was calculated according to the Null Model, which assumes that all mutational effects are additive.

[e] The observed total change was calculated by comparing the actual activity of the evolutionary end point (AE or TriA) to that of the starting point (*wt*PTE or AtzA, respectively).

The activity pattern of the mutational effects observed in the newly evolved specialist enzyme, AE, also opposes the robustness model. As a complete activity switch has occurred, the newly acquired arylesterase activity is now, in effect, the native activity and phosphotriesterase the promiscuous activity. The null-model predicts a 10^10^-fold decrease in the arylesterase activity, which is a larger decrease than that observed when all 26 mutations are reverted together (10^5^-fold). In contrast, the predicted change in phosphotriesterase activity is similar to the observed change (~10^5^-fold increase). Therefore, rather than acquiring robustness, the newly evolved enzyme becomes more plastic in its main activity (arylesterase in AE) during the adaptive process. In summary, these results show that both specialist enzymes, *wt*PTE and AE, do not acquire the properties proposed by the robustness model.

### The weak trade-off is caused by epistasis

**Fig 4:**
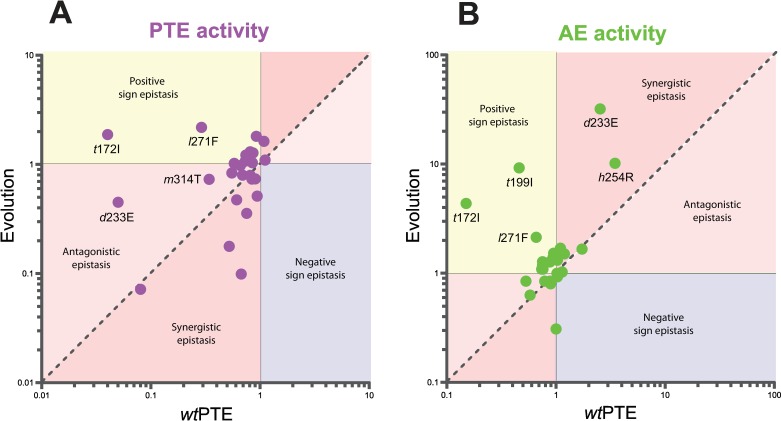
Epistasis between mutations in the background of *wt*PTE and upon their occurence in the evolution. (A) PTE activity. (B) AE activity. Note the difference in scale between the two panels. Activities are given relative to the respective parent background. Mutations causing a >1.3-fold change compared to the parent mutant are considered non-neutral. A student T-test was performed and P-values compared to each parent (**S1 and S2 Tables**) and p-values for the effect of each mutation (**S5 Table**) in the two backgrounds were calculated. Note that the effect of *t*199I and *a*80V on paraoxon hydrolysis as well as the effect of *a*49V and *e*144V on 2NH hydrolysis are statistically not significantly different between the two backgrounds. Selected mutations are labelled and amino acids found in *wt*PTE shown in lower-case italics.

At first glance, it seems paradoxical that during the early rounds of the trajectory, only weak trade-offs are observed despite the fact that the original activity is much more plastic than the new activity in the background of *wt*PTE in terms of individual mutational effects. Comparing the individual mutations in the three different backgrounds revealed extensive epistasis during the functional transition, *i*.*e*., changes in the effect of mutations depending on the genetic background in which they occur. In general, synergistic epistasis between deleterious mutations is considered to be a feature of mutational robustness; proteins can tolerate individual mutations but the accumulation of multiple mutations is more detrimental than expected from each single mutational effect [37, 38]. However, we find that antagonistic and even positive sign epistasis underlies the relatively mild reduction of phosphotriesterase activity. For example, 15 were initially deleterious for phosphotriesterase activity in the background of *wt*PTE. Out of these 15 mutations, seven mutations became only moderately deleterious or neutral at their actual point of occurrence in the evolutionary trajectory and two mutations reversed their effect from negative to positive (**Fig 3A and 3B** and **Fig 4A**, **S5 Table**). This reinforces our observations that the native activity of PTE is not robust to mutational perturbations. On the other hand, the significant increase in arylesterase activity during the trajectory was enabled through synergistic and positive sign epistasis. 22 of 26 mutations were initially deleterious or neutral for AE activity in the *wt*PTE background. However, 10 of these mutations became favourable during the evolution due to accumulation of earlier mutations: six (neutral) mutations increased their positive effect on arylesterase activity and four mutations changed their effect from deleterious to positive (**Fig 3C and 3D** and **Fig 4B**, **S5 Table**). Thus, the evolution of arylesterase activity appears to depend on early permissive mutations, *i*.*e*., on early mutations that enable or enhance the effect of later mutations. While the molecular basis of epistasis remains to be elucidated in most cases, our previous work offers explanations for several mutational interactions in the evolution of PTE [29, 33, 39, 40]. Here, we would like to focus solely on the fact that the trajectory exhibits weak trade-offs in the early stages of evolution, due to opposite epistatic effects in the original and new activities (one synergistic, one antagonistic); the new activity is not sensitive to individual mutations whereas the original activity is highly sensitive.

### Characterization of a random mutational library confirms the lack of mutational robustness of the native activity

**Fig 5:**
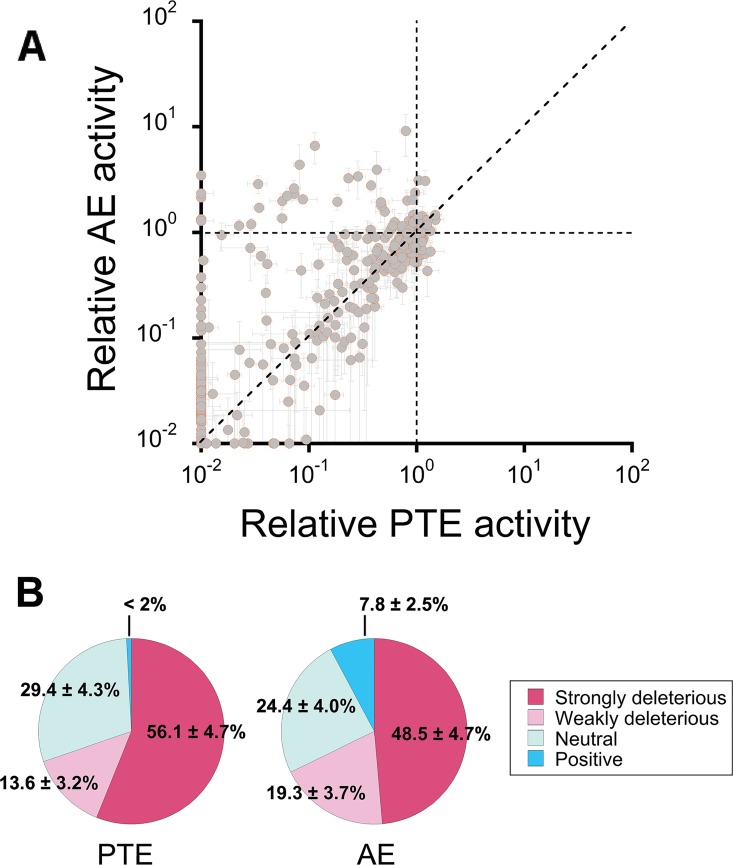
Functional analysis of a random mutant library. (A) Changes in phosphotriesterase (native; PTE) and arylesterase (promiscuous; AE) activities among variants from a trinucleotide substitution library. The enzymatic activities for each variant (shown as grey dots) are plotted relative to those of *wt*PTE. Data are averages of triplicate values from three independent experiments and error bars represent +/- 1 SEM. (B) Distribution of the mutational impact on phosphotriesterase and arylesterase activities. Mutations are classified as strongly deleterious (>2-fold activity decrease relative to *wt*PTE), weakly deleterious (2-fold—1.3-fold decrease), neutral (<1.3-fold change), and positive (>1.3-fold increase). Frequencies are indicated with their corresponding 95% confidence intervals.

The above analysis includes only 26 adaptive mutations that collectively switch function and, therefore, may be biased. A random sample of mutants not selected for an activity increase may show a different distribution. To test this possibility, we expanded our analysis to several hundred random mutations by generating a *wt*PTE trinucleotide substitution library and randomly selecting > 400 variants that were subsequently assayed for both phosphotriesterase and arylesterase activity. The functional effects of these random mutations support the observations made for the subset of 26 mutations (**Fig 5, S6 Table**); ~56% of the mutations were strongly deleterious (*i*.*e*., >2-fold activity decrease) for phosphotriesterase activity and ~49% for arylesterase activity. Therefore, the native activity is evidently not more robust than the promiscuous activity. The overall negative tendency of the mutational effects between phosphotriesterase and arylesterase activities was supported by statistical tests (*p*<0.0001 for Kolmogorov–Smirnov test and Wilcoxon signed-rank test, **S7 Table** and **S1 Fig**). The same trend was observed among the ~8% of mutations that increased arylesterase activity, *i*.*e*., adaptive mutations (>1.3-fold activity increase): The majority of mutations (>60%) were strongly deleterious for phosphotriesterase activity (>2-fold decrease). By contrast, a much smaller fraction of mutations were neutral (~29%) or positive (~5%). This indicates that, in general, the majority of mutations cause a strong trade-off between the two activities **(S1**and **S2 Figs, S7 Table**), *i*.*e*., in this example, mutational robustness cannot explain the weak trade-offs observed during laboratory evolution.

### Evolutionary transition between AtzA and TriA

**Fig 6:**
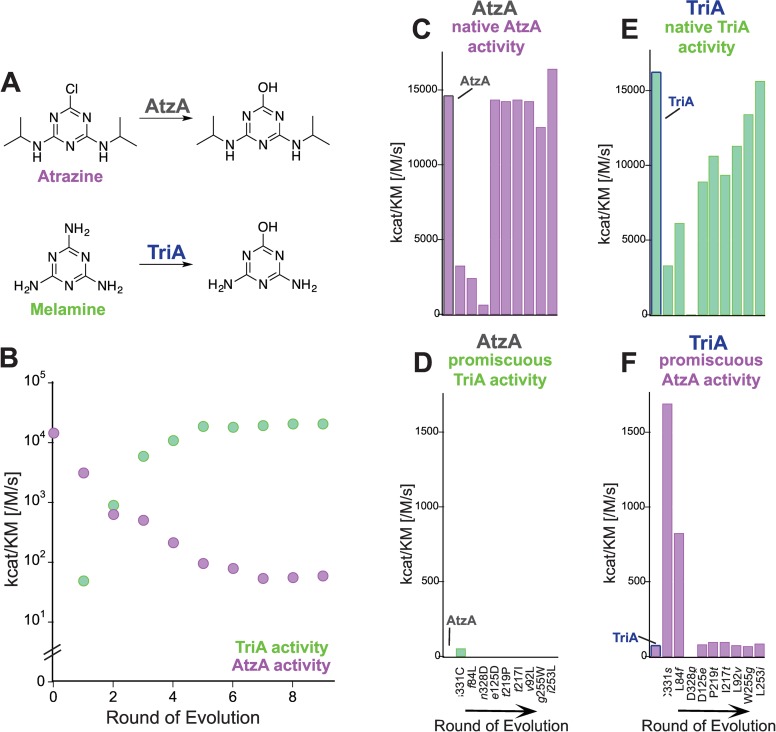
Evolution from AtzA to TriA (adapted from reference [34]). (A) AtzA catalyzes the dechlorination of atrazine (AtzA activity). TriA catalyzes the deamination of melamine (TriA activity). TriA catalyzed the dechlorination reaction promiscuously. Deamination by AtzA could not be detected. (B) A possible uphill evolutionary trajectory from AtzA to TriA determined by Noor et al. In each round of evolution, a single point mutation was added in the order shown in (C)—(F) (see also **S8 Table**). (C)—(F) Effect of all single point mutations separating AtzA and TriA (**S9 and S10 Tables**). (C) Effect of mutations in the AtzA background on AtzA activity and (D) TriA activity. (E) Effect of mutations in the TriA background on TriA activity and (F) AtzA activity. Activities are expressed as *k*_*cat*_/*K*_*M*_ values. Relative activities could not be calculated because several variants do not have detectable activity. Amino acids found in AtzA are shown in lower-case italics.

In order to examine whether the observed mutational effects are unique to PTE or a more general property of promiscuous enzymes, we searched for literature examples containing similar data sets, consisting of (i) an evolutionary trajectory leading to a complete switch between native and promiscuous activity and (ii) mutational data in the background of both the starting and end point as well as during the trajectory. Only one example met both criteria: Noor and co-workers describe the natural evolutionary transition between AtzA and TriA, two recently evolved enzymes involved in degrading xenobiotic compounds [41, 42]. AtzA catalyzes the dechlorination of atrazine, and TriA catalyzes the deamination of melanine (**Fig 6A**). Despite specificity for their respective substrates, the two enzymes differ in only 9 amino acids. Noor and coworkers reconstructed the most likely historical transition from AtzA to TriA to reveal how the stepwise accumulation of these nine mutations could follow an uphill trajectory with a >10^4^-fold increase in TriA activity (**Fig 6B, S8 Table**) [34]. Mirroring the observations made for the evolution of PTE, the TriA trajectory exhibited weak trade-offs. We analyzed the effects of each mutation in the background of AtzA and TriA (**S9 and S10 Tables**), and found a pattern similar to that observed for PTE. Functional mutations in the early stages of evolution play a permissive role for later mutations: none of the later mutations are able to increase TriA activity in the starting point, AtzA, but they become favorable over the course of evolution due to interaction with the early mutations. The weak trade-off does not result from robustness in the native dechlorination activity of AtzA: the predicted effect of introducing all nine mutations into AtzA based on the null-model (770-fold) exceeds the observed decrease over the trajectory (240-fold, **Fig 6C and 6D, Table 1**). Similarly, deamination, the native function of TriA, is not robust in this background: the mutation N328D causes a complete loss of function in TriA (>10^4^-fold reduction), but an only 8-fold change when it occurs in the trajectory (**Fig 6E and 6F, Table 1**). Again, these observations do not support the robustness model whereas the proposed evolutionary trajectory followed the typical weak trade-offs between the two activities.

## Discussion

In this work, we analyzed two cases of enzyme evolution–one from the laboratory and one from nature–and showed that the robustness model for weak trade-offs cannot explain the observed evolutionary trajectories. In both cases, trade-offs were weak in the early steps of evolution. Furthermore, this effect can be observed in many other directed evolution studies where enzymes do not display mutational robustness in their native activity, and the native activity can be more sensitive to mutations than the promiscuous activity [43–45]. It is difficult to say whether the two examples described here represent a general feature of all enzymes. Specifically, both enzymes are products of recent evolution towards xenobiotic degradation, and therefore cannot have experienced long periods of neutral genetic drift [46, 47]. It has been postulated that mutational robustness can be a result of extensive drift [48–51]. This is because impaired variants will be purged from a population upon mutation, while highly mutable variants would become enriched. It is therefore possible that well-conserved enzymes with a longer evolutionary history exhibit a higher degree of robustness; extensive mutational data, however, is not currently available for such enzymes. Irrespective of whether our findings can be extended to all enzymes, robustness in the native activity is not a prerequisite for weak trade-offs between new and original activities. To date, these factors are seen as inextricably linked, and our observations alter this view.

Mutational robustness has recently been considered an essential property of biological systems [52–55]. Robustness has been observed on various levels, *e*.*g*., in regulatory networks [56, 57], metabolic pathways [58, 59] and proteins [37, 60, 61]. On the protein level, robustness can be seen in terms of protein stability and function; the relationship between protein stability and robustness has been well established [61–64]. Because most mutations reduce protein stability, proteins are maintained above the stability threshold, resulting in mutational robustness (or tolerance to reductions in stability) and thus evolvability [62]. In contrast, a connection between mutational robustness and protein function (e.g., enzymatic activity) has been discussed but not conclusively established. For instance, one could argue that a high level of native catalytic activity causes a protein to be more vulnerable, rather than less, to mutational perturbations directly opposing the robustness model. In order to be highly efficient catalysts, an enzyme’s active site architecture is evolutionarily fine-tuned to contain effective and well-positioned catalytic amino acid groups and/or particular dynamics [65–69], so any deviation from this optimum through mutations may generally be deleterious. Therefore, enzymes might not be able to simultaneously acquire high efficiency and mutational robustness. In accordance with this idea, we detect antagonistic epistasis in PTE’s native activity: individual mutations are more deleterious on their own than when they are combined. A recent study by Bank *et al*. that explored >1000 double mutants of yeast Hsp90 also found that antagonistic epistasis in the native protein function was frequent (46% antagonistic vs. 1.8% of synergistic) [70]. Promiscuous secondary activities, on the other hand, may be more tolerant (or less sensitive) to mutations as the active site architecture has not been optimized to perform this particular reaction and the catalytic effects are less sophisticated [3]. Recent studies indicate that mutational pathways to improve promiscuous functions are highly restricted [29, 71–75]. In addition, a systematic study showed that the evolution of a promiscuous activity is predominantly driven by synergistic, positive epistasis: mutations that occurred later along the laboratory trajectory owed their positive effect to earlier, permissive mutations [76].

Given the strong experimental evidence arguing against the universal application of the mutational robustness hypothesis, the question of how the frequent observation of weak trade-offs can be explained arises. Our results are consistent with the proposal that a strong selection pressure for the new function causes a bias towards weak trade-offs. In typical adaptive evolution, in particular evolution in the laboratory, the only selection criterion is to increase the target function, but no selection pressure is applied for or against the original function. Under such conditions, mutations that confer the largest increase in the new activity are strongly favoured, whether or not the original activity changes. Thus, the accumulation of highly adaptive mutations results in a significant and predictable increase in the selected activity, but the concurrent reduction in the native activity is effectively stochastic and random. In other words, the extent of any such reduction is not correlated with any such increase because it is irrelevant for selection whether and by how much the old function changes, making evolution initially biased towards weak trade-offs. Strong functional trade-offs have also been observed, but much less frequently [43–45]. To develop a deeper understanding of what causes varying degrees of trade-offs, a mechanistic exploration at the molecular level is necessary. Ideally, a functional role needs to be assigned to each residue for each activity, while also taking into account epistasis.

Regardless of whether mutational robustness in the native activity is present or not, adaptation to a new function has been frequently shown to be accompanied by weak trade-offs in laboratory evolution experiments. Extrapolated to a scenario in natural evolution, this type of trajectory would reduce the selection pressure on early gene duplication because a high level of the original function can be maintained with just one copy of the gene [6]. It remains unclear, however, how specialization can be achieved when the only selection pressure is to increase the new function. Some directed evolution studies propose that trade-offs can become stronger as evolution proceeds [33, 77], but many other experiments have resulted in bi-functional, generalist enzymes [4, 18–31]. The high functional specificity of extant enzymes may only have emerged after extended selection and/or extensive genetic drift. Moreover, in nature, selection to reduce the original function may exist. For example, the native and new substrate may compete for the active site of the enzyme, causing cross inhibition. In such a case, gene duplication followed by prompt specialization (rapid trade-offs) seems a likely scenario [78]. Nevertheless, each of these outcomes would be consistent with the common, though not universal, phenomenon of weak adaptive trade-offs that have been observed.

## Methods

### Construction and characterization of single mutants

Mutants were constructed by site-directed mutagenesis as described in the QuikChange Site-Directed Mutagenesis manual (Agilent).

### Kinetic characterization of PTE variants

To determine relative initial rates in lysate, the variants selected over the evolution and the additional single mutants were assayed in parallel as follows. *E*. *coli* BL21 (DE3) cells containing a pGro7 plasmid (for co-expression of GroEL/ES) were transformed with pET-Strep plasmids containing PTE variants (or the *wt*PTE TriNEx library) and plated on LB agar containing 100 μg/mL ampicillin (amp, pET-Strep plasmid) and 34 μg/mL chloramphenicol (cam, pGro7 plasmid). Single colonies were picked and grown overnight in 96 deep-well plates containing LB medium with 100 μg/mL amp and 34 μg/mL cam at 30°C with shaking. These overnight cultures were used to inoculate (at 1:20 dilution) LB medium containing amp, cam, and 200 μM ZnCl_2_ in 96 deep-well plates. In addition, the medium contained 0.2% (w/v) arabinose to induce GroEL/ES expression. Cells were grown at 37°C with shaking for about 2–3 h until the OD_600_ reached 0.6–1.0, at which point IPTG (1 mM final) was added to induce over-expression of the PTE variants. Following a 2 h incubation at 30°C, the OD_600_ was measured again, and cells were pelleted and stored at -80°C for at least 1 h. Cells were resuspended in lysis buffer (50 mM Tris-HCl pH 7.5, 100 μg/mL lysozyme, 0.5 units/mL benzonase, and 0.1% Triton X-100). After 30 minutes of lysis, lysates were clarified by centrifugation, diluted according to the level of activity, and assayed for enzymatic activity in 200 μL reactions containing 180 μM of the substrates paraoxon (Sigma) or 2NH (in the presence of 180 μM FAST Red, both Sigma). Hydrolysis product formation was followed at 500 nm for the naphtholate–FAST Red complex and at 405 nm in the case of *p-*nitrophenolate. Initial rates were normalized to cell density using the OD_600_ values. Cells were grown in at least duplicate. The experiment was repeated and the average change relative to the respective parent variant and the standard deviation were determined.

### Construction of a substitution variant library from wtPTE

A library of random single variants was generated from *wt*PTE using trinucleotide exchange (TriNEx [79]; see **S1 Text** for a detailed description of the procedure). Briefly, a synthetic *wt*PTE gene containing no MlyI restriction site was designed, synthesized (GenScript, NJ, USA) and cloned in pID-Tet using NcoI and HindIII. A transposon insertion library was then generated in this gene with TransDel (a MuDel-like engineered transposon). The plasmid pool corresponding to this library was digested with MlyI to excise out TransDel and subsequently insert the SubsNNN DNA cassette (corresponding to SubSeqNNN in the original publication). The plasmid pool corresponding to the resulting SubsNNN insertion library was digested with MlyI to remove SubsNNN and the resulting linear plasmid was self-circularized by ligation. After transformation, the plasmids corresponding to the resulting TriNEx library of *wt*PTE were purified from the pooled bacterial colonies. Finally, the library was excised by NcoI/HindIII double digestion and subcloned into pET-Strep vector.

### Kinetic characterization of the substitution variant library

A total of 435 variants were screened for 2NH and paraoxonase activity in triplicates in crude cell lysates as described above (“**Kinetic characterization of PTE variants**”). The experiment was repeated twice (giving a total of three independent sets of bacterial growth/protein expression/activity screen) and the average change relative to the respective parent variant and the standard error of the mean were determined.

## Supporting Information

S1 Table Overview of the 26 mutations obtained in the directed evolution experiment. (PDF)

S2 Table Effect of mutations in the *wt*PTE background on paraoxon and 2NH hydrolysis in cell lysate. (PDF)

S3 Table Effect of mutations in the AE background on paraoxon and 2NH hydrolysis in cell lysate. (PDF)

S4 Table Effect of mutations in the evolution on paraoxon and 2NH hydrolysis in cell lysate. (PDF)

S5 Table Comparison of the effect of mutations in the evolution and in *wt*PTE. (PDF)

S6 Table Functional analysis of a random library of *wt*PTE variants. (PDF)

S7 Table Statistical parameters derived from the functional characterization of the random library of *wt*PTE variants. (PDF)

S8 Table Overview of the 9 mutations in the evolution from AtzA to TriA (adapted from reference [4]). (PDF)

S9 Table Effect of mutations on atrazine dechlorination (adapted from reference [4]). (PDF)

S10 Table Effect of mutations on melamine deamination (adapted from reference [4]). (PDF)

S1 Fig Distribution of the difference of the log-transformed PTE and AE relative activities for each variant (n = 435). (PDF)

S2 Fig Distribution of the effect of single trinucleotide substitutions on the native phosphotriesterase activity depending on their effect on the promiscuous arylesterase activity. (PDF)

S1 Text Detailed Methods. (PDF)
