# Supplementary material for: Functional Trade-Offs in Promiscuous Enzymes Cannot Be Explained by Intrinsic Mutational Robustness of the Native Activity
Source: PLoS Genet. 2016 Oct 7;12(10):e1006305. doi: 10.1371/journal.pgen.1006305 (PMC5065130; doi:10.1371/journal.pgen.1006305)
Supplement: S1 Table — (PDF) [file pgen.1006305.s001.pdf]

Supporting Information for Kaltenbach at al.,

Functional trade-offs in promiscuous enzymes cannot be explained by intrinsic mutational robustness of the native activity

S1 Table. Overview of the 26 mutations obtained in the directed evolution experiment. Mutations are shown relative to *wt*PTE (GenBank accession number KJ680379) with lower case italics denoting the amino acid found in *wt*PTE. Note that *wt*PTE was obtained in previous screens for improved expression levels in *E. coli* and contains six mutations relative to the naturally occurring PTE (I106L, F132L, K185R, D208G, R319S) [1, 2]. The following mutations occurred in individual variants, but were not fixated after DNA shuffling. R7a: *a*204G, R7c: *a*102V, R19: *a*78T, *v*143A, *t*311A.

Mutations

Round	Variant	*h254*	*d233*	*f306*	*i274*	*t172*	*s269*	*m138*	*t199*	*l272*	*a80*	*s111*	*a204*	*l130*	*l271*	*a49*	*k77*	*l140*	*i313*	*s137*	*q180*	*t45*	*e144*	*m314*	*i341*	*s102*	*v176*
0	*wt*PTE																										
1	R1	R																									
2	R2a	R		L																							
2	R2b	R	E																								
3	R3	R		L	S																						
4	R4	R	E	L	S																						
5	R5a	R	E	L	S	I																					
5	R5b	R	E	L	S		T																				
6	R6	R	E	L	S	I	T																				
7	R7a[a]	R	E	I	S	I	T	I																			
7	R7b	R	E	I	S	I	T																				
7	R7c[b]	R	E	I	S	I	T		I																		
8	R8	R	E	I	S	I	T	I	I																		
9	R9	R	E	I	S	I	T	I	I	M																	
10	R10	R	E	I	S	I	T	I	I	M	V																
11	R11a	R	E	I	S	I	T	I	I	M	V	R															
11	R11b	R	E	I	S	I	T	I	I	M	V		G														
12	R12	R	E	I	S	I	T	I	I	M	V	R	G														
13	R13a	R	E	I	S	I	T	I	I	M	V	R	G		F												
13	R13b	R	E	I	S	I	T	I	I	M	V	R	G	V													
14	R14	R	E	I	S	I	T	I	I	M	V	R	G	V	F												
18	R18	R	E	I	S	I	T	I	I	M	V	R	G	V	F	V	E	M	F								
19	R19[c]	R	E	I	S	I	T	I	I	M	V	R	G	V	F	V	E	M	F	T	H						
20	R20	R	E	I	S	I	T	I	I	M	V	R	G	V	F	V	E	M	F	T	H	A	V	T	T		
21	R21	R	E	I	S	I	T	I	I	M	V	R	G	V	F	V	E	M	F	T	H	A	V	T	T	T	
22	AE	R	E	I	S	I	T	I	I	M	V	R	G	V	F	V	E	M	F	T	H	A	V	T	T	T	M

1. Roodveldt C, Tawfik DS. Shared promiscuous activities and evolutionary features in various members of the amidohydrolase superfamily. Biochemistry. 2005;44(38):12728-36.

2. Tokuriki N, Jackson CJ, Afriat-Jurnou L, Wyganowski KT, Tang R, Tawfik DS. Diminishing returns and tradeoffs constrain the laboratory optimization of an enzyme. Nature Communications. 2012;3:1257.
