# Supplementary material for: Functional Trade-Offs in Promiscuous Enzymes Cannot Be Explained by Intrinsic Mutational Robustness of the Native Activity
Source: PLoS Genet. 2016 Oct 7;12(10):e1006305. doi: 10.1371/journal.pgen.1006305 (PMC5065130; doi:10.1371/journal.pgen.1006305)
Supplement: S2 Table — (PDF) [file pgen.1006305.s002.pdf]

Functional trade-offs in promiscuous enzymes cannot be explained by intrinsic mutational robustness of the native activity

S2 Table. Effect of mutations in the *wt*PTE background on paraoxon and 2NH hydrolysis in cell lysate.

Mutation[a]	Round[b]	Paraoxon		2NH	
		relative activity[f]	T-test p-value[g]	relative activity[f]	T-test p-value[g]
*h*254R	1	0.1±0.02	5.9×10^{-7}	3.5±0.2	6.2×10^{-4}
*d*233E	2/4	0.1±0.02	6.3×10^{-9}	2.5±0.5	2.0×10^{-4}
*f*306I	2-8[c]	0.02±0.01	5.3×10^{-8}	11±2	2.1×10^{-7}
*i*274S	3/4	0.9±0.3	0.24	1.0±0.1	0.70
*t*172I	5/6	0.3±0.1	1.3×10^{-6}	0.1±0.1	5.6×10^{-8}
*s*269T	5/6	0.9±0.1	0.17	1.0±0.1	0.54
*m*138I	7/8	0.8±0.3[h]	0.10	1.0±0.1	0.99
*t*199I	7/8	1.1±0.5	0.67	0.5±0.1	1.2×10^{-5}
*l*272M	9	0.6±0.1	9.5×10^{-5}	0.8±0.1	2.1×10^{-3}
*a*80V	10	0.9±0.1	0.04	1.1±0.1	0.14
*s*111R	11/12	0.7±0.1	0.01	0.7±0.2	0.03
*a*204G	11/12	0.7±0.1	4.6×10^{-3}	0.7±0.1	1.3×10^{-3}
*l*130V	13/14	0.7±0.2	0.02	0.7±0.1	3.7×10^{-3}
*l*271F	13/14	0.04±0.01	4.2×10^{-10}	0.7±0.2	1.2×10^{-3}
*a*49V	18[d]	1.1±0.1	0.23	0.9±0.02	0.07
*k*77E	18[d]	0.7±0.2	2.5×10^{-3}	0.9±0.1	0.38
*l*140M	18[d]	0.9±0.2	0.35	1.7±0.2	1.9×10^{-5}
*i*313F	18[d]	0.7±0.2	3.0×10^{-3}	1.2±0.2	0.03
*s*137T	19/20[e]	0.8±0.3	0.21	0.8±0.2	0.04
*q*180H	19/20[e]	0.5±0.1	1.3×10^{-4}	0.6±0.1	2.9×10^{-4}
*t*45A	19/20[e]	0.8±0.2	0.04	0.9±0.1	0.42
*e*144V	19/20[e]	0.6±0.2	7.6×10^{-4}	0.5±0.2	6.3×10^{-4}
*m*314T	19/20[e]	0.3±0.1	4.8×10^{-6}	1.1±0.3	0.26
*i*341T	19/20[e]	0.8±0.1	0.01	0.9±0.1	0.03
*s*102T	21[e]	0.9±0.2	0.51	0.9±0.2	0.12
*v*176M	22[e]	0.6±0.2	1.3×10^{-3}	1.0±0.2	0.69

[a] Amino acids present in *wt*PTE are shown in lower-case italics.
[b] When two rounds are shown, the first number indicates the initial round of appearance and the second number indicates the round of fixation after DNA shuffling.
[c] In the forward evolution, *f*306 was initially mutated to L in round 2 (fixated after DNA shuffling in round 4). In round 7, L306 was further mutated to I and fixated after DNA shuffling in round 8. Note that therefore, the effect of *f*306I in the evolution could not be determined.
[d] In rounds 15-17, no significantly improved variants could be identified. Therefore, a pool of variants was taken into the next round, yielding an improved variant in round 18. A detailed description of the directed evolution experiment can be found in [1].
[e] In rounds 19-22, variants were screened for a reduction in paraoxon hydrolysis and maintenance of 2NH hydrolysis. A detailed description of the directed evolution experiment can be found in [1, 2].
[f] Cells were grown in at least duplicate and lysates sufficiently diluted (~1-10,000-fold) to determine initial rates v_0 of paraoxon and 2NH hydrolysis at a substrate concentration of 200 μM, normalized to cell density, and corrected for the dilution factor. This experiment was repeated twice and the average

change of each variant relative to *wt*PTE (dimensionless ratio of the v_0) and the standard deviation were determined.
[g] A student t-test was performed to obtain p-values. Only mutants with an average >1.3-fold difference from the respective parent mutant AND a p-value <0.05 are considered significant. The cut-off of 1.3-fold was applied because only variants that differ by at least this amount from their respective parent could reliably be identified in our screening system. Non-significant values are underlined.
[h] Note that *m*138I has a >1.3 fold effect on PTE activity but a non-significant p-value.

1. Tokuriki N, Jackson CJ, Afriat-Jurnou L, Wyganowski KT, Tang R, Tawfik DS. Diminishing returns and tradeoffs constrain the laboratory optimization of an enzyme. Nature Communications. 2012;3:1257.
2. Kaltenbach M, Jackson CJ, Campbell EC, Hollfelder F, Tokuriki N. Reverse evolution leads to genotypic incompatibility despite functional and active-site convergence. Elife. 2015;4.
