# Supplementary material for: Functional Trade-Offs in Promiscuous Enzymes Cannot Be Explained by Intrinsic Mutational Robustness of the Native Activity
Source: PLoS Genet. 2016 Oct 7;12(10):e1006305. doi: 10.1371/journal.pgen.1006305 (PMC5065130; doi:10.1371/journal.pgen.1006305)
Supplement: S3 Table — (PDF) [file pgen.1006305.s003.pdf]

Functional trade-offs in promiscuous enzymes cannot be explained by intrinsic mutational robustness of the native activity

S3 Table. Effect of mutations in the AE background on paraoxon and 2NH hydrolysis in cell lysate.

Mutation[a]	Round[b]	Paraoxon relative activity[f]	Paraoxon T-test p-value[g]	2NH relative activity[f]	2NH T-test p-value[g]
R254*h*	1	<0.1[h]	/	(1.9±0.5)×10^{-5}	2.3×10^{-4}
E233*d*	2/4	4.6±0.3	7.8×10^{-11}	0.2±0.1	3.4×10^{-6}
I306*f*	2-8[c]	1.8±0.2	9.3×10^{-6}	0.02±0.01	2.2×10^{-6}
S274*i*	3/4	1.0±0.1	0.84	0.8±0.3	0.28
I172*t*	5/6	2.2±0.6	4.5×10^{-4}	0.4±0.1	1.1×10^{-4}
T269*s*	5/6	1.0±0.1	0.74	1.0±0.4	0.83
I138*m*	7/8	1.7±0.2	9.2×10^{-5}	1.0±0.1	0.87
I199*t*	7/8	0.6±0.1	4.1×10^{-4}	0.4±0.2	7.5×10^{-4}
M272*l*	9	3.1±0.7	3.1×10^{-5}	0.4±0.1	2.4×10^{-4}
V80*a*	10	0.8±0.2	0.08	0.8±0.3	0.20
R111*s*	11/12	0.9±0.1	0.27	0.8±0.2	0.14
G204*a*	11/12	1.3±0.2	0.02	0.9±0.3	0.34
V130*l*	13/14	3.6±0.2	1.6×10^{-8}	1.1±0.4	0.77
F271*l*	13/14	7.2±0.6	7.6×10^{-9}	0.2±0.1	9.2×10^{-6}
V49a	18[d]	1.2±0.2	0.05	1.1±0.5	0.80
E77*k*	18[d]	1.2±0.1	0.10	1.1±0.4	0.57
M140*l*	18[d]	0.7±0.1	0.01	0.7±0.2	0.04
F313*i*	18[d]	8.0±1.0	9.3×10^{-8}	0.6±0.1	2.6×10^{-3}
T137*s*	19/20[e]	1.5±0.04	7.4×10^{-5}	0.9±0.4	0.67
H180*q*	19/20[e]	2.8±0.3	1.4×10^{-6}	1.1±0.2	0.31
A45*t*	19/20[e]	1.0±0.1	0.56	1.0±0.3	0.83
V144*e*	19/20[e]	1.3±0.1	0.01	1.1±0.3	0.40
T314*m*	19/20[e]	1.5±0.1	3.4×10^{-4}	1.0±0.3	0.94
T341*i*	19/20[e]	1.4±0.1	3.2×10^{-3}	1.3±0.4[i]	0.17
T102*s*	21[e]	2.1±0.3	3.1×10^{-5}	1.1±0.2	0.54
M176*v*	22[e]	0.9±0.1	0.31	0.8±0.2	0.11

[a] Amino acids present in *wt*PTE are shown in lower-case italics.

[b] When two rounds are shown, the first number indicates the initial round of appearance and the second number indicates the round of fixation after DNA shuffling.

[c] In the forward evolution, *f*306 was initially mutated to L in round 2 (fixated after DNA shuffling in round 4). In round 7, L306 was further mutated to I and fixated after DNA shuffling in round 8. Note that therefore, the effect of *f*306I in the evolution could not be determined.

[d] In rounds 15-17, no significantly improved variants could be identified. Therefore, a pool of variants was taken into the next round, yielding an improved variant in round 18. A detailed description of the directed evolution experiment can be found in [1].

[e] In rounds 19-22, variants were screened for a reduction in paraoxon hydrolysis and maintenance of 2NH hydrolysis. A detailed description of the directed evolution experiment can be found in [1, 2].

[f] Cells were grown in at least duplicate and lysates sufficiently diluted (~1-10,000-fold) to determine initial rates v_0 of paraoxon and 2NH hydrolysis at a substrate concentration of 200 μM, normalized to

cell density, and corrected for the dilution factor. This experiment was repeated twice and the average change of each variant relative to AE (dimensionless ratio of the v_0) and the standard deviation were determined.

[g] A student t-test was performed to obtain p-values. Only mutants with an average >1.3-fold difference from the respective parent mutant AND a p-value <0.05 are considered significant. The cut-off of 1.3-fold was applied because only variants that differ by at least this amount from their respective parent could reliably be identified in our screening system. Non-significant values are underlined.

[h] The initial rate of paraxon hydrolysis was too low to be determined, but at least 10-fold reduced compared to AE.

[i] Note that T341*i* has a >1.3 fold effect on AE activity but a non-significant p-value.

1. Tokuriki N, Jackson CJ, Afriat-Jurnou L, Wyganowski KT, Tang R, Tawfik DS. Diminishing returns and tradeoffs constrain the laboratory optimization of an enzyme. Nature Communications. 2012;3:1257.

2. Kaltenbach M, Jackson CJ, Campbell EC, Hollfelder F, Tokuriki N. Reverse evolution leads to genotypic incompatibility despite functional and active-site convergence. Elife. 2015;4.
