# Supplementary material for: Functional Trade-Offs in Promiscuous Enzymes Cannot Be Explained by Intrinsic Mutational Robustness of the Native Activity
Source: PLoS Genet. 2016 Oct 7;12(10):e1006305. doi: 10.1371/journal.pgen.1006305 (PMC5065130; doi:10.1371/journal.pgen.1006305)
Supplement: S4 Table — (PDF) [file pgen.1006305.s004.pdf]

Functional trade-offs in promiscuous enzymes cannot be explained by intrinsic mutational robustness of the native activity

S4 Table. Effect of mutations in the evolution on paraoxon and 2NH hydrolysis in cell lysate.

Mutation[a]	Round[b]	Paraoxon relative activity[f]	Paraoxon T-test p-value[g]	2NH relative activity[f]	2NH T-test p-value[g]	Calculation
*h*254R	1	0.1±0.02	3.0×10^{-5}	10±5	0.01	R1 / *wt*PTE
*d*233E	2/4	0.5±0.04	0.01	32±3	8.2×10^{-7}	R2b / R1
*f*306I	2-8[c]	/	/	/	/	/
*i*274S	3/4	1.0±0.2	0.78	1.0±0.2	0.98	R3 / R2a
*t*172I	5/6	2.2±0.4	4.4×10^{-3}	4.4±1.3	1.9×10^{-3}	R6 / R5b
*s*269T	5/6	1.8±0.3	4.4×10^{-3}	1.3±0.4[g]	0.16	R6 / R5a
*m*138I	7/8	0.4±0.1	2.0×10^{-3}	0.3±0.1	3.9×10^{-6}	R8 / R8+I138*m*
*t*199I	7/8	1.6±0.6[h]	0.06	9.3±2.4	1.3×10^{-4}	R8 / R8+I199*t*
*l*272M	9	0.5±0.2	0.01	1.1±0.3	0.60	R9 / R8
*a*80V	10	1.3±0.4	0.19	1.7±0.3	4.2×10^{-3}	R10 / R9
*s*111R	11/12	0.8±0.2	0.03	1.3±0.1	0.02	R12 / R11b
*a*204G	11/12	1.2±0.2	0.10	1.1±0.1	0.55	R12 / R11a
*l*130V	13/14	1.1±0.1	0.68	1.2±0.1	0.15	R14 / R13a
*l*271F	13/14	1.9±0.1	3.0×10^{-6}	2.1±0.2	3.7×10^{-5}	R14 / R13b
*a*49V	18[d]	1.1±0.01	0.14	1.3±0.2	0.19	R18 / R18+V49*a*
*k*77E	18[d]	1.0±0.1	0.58	1.4±0.2	0.04	R18 / R18+E77*k*
*l*140M	18[d]	0.7±0.1[h]	0.41	1.7±0.3	3.7×10^{-3}	R18 / R18+M140*l*
*i*313F	18[d]	0.1±0.01	7.8×10^{-9}	1.5±0.3	0.02	R18 / R18+F313*i*
*s*137T	19/20[e]	0.7±0.1	1.1×10^{-3}	0.9±0.2	0.38	R20 / R20+T137*s*
*q*180H	19/20[e]	0.2±0.02	4.9×10^{-5}	0.6±0.02	0.01	R20 / R20+H180*q*
*t*45A	19/20[e]	1.3±0.1[h]	0.72	1.5±0.3	0.04	R20 / R20+A45*t*
*e*144V	19/20[e]	0.8±0.1	0.04	0.8±0.2	0.27	R20 / R20+V144*e*
*m*314T	19/20[e]	0.7±0.1	1.0×10^{-3}	1.0±0.2	0.81	R20 / R20+T314*m*
*i*341T	19/20[e]	0.8±0.1	3.3×10^{-3}	0.8±0.2	0.51	R20 / R20+T341*i*
*s*102T	21[e]	0.5±0.1	1.7×10^{-4}	0.9±0.2	0.31	R21 / R20
*v*176M	22[e]	1.0±0.04	0.72	0.9±0.1	0.57	AE / R21

[a] Amino acids present in *wt*PTE are shown in lower-case italics.
[b] When two rounds are shown, the first number indicates the initial round of appearance and the second number indicates the round of fixation after DNA shuffling.
[c] In the forward evolution, f306 was initially mutated to L in round 2 (fixated after DNA shuffling in round 4). In round 7, L306 was further mutated to I and fixated after DNA shuffling in round 8. Note that therefore, the effect of *f*306I in the evolution could not be determined.
[d] In rounds 15-17, no significantly improved variants could be identified. Therefore, a pool of variants was taken into the next round, yielding an improved variant in round 18. A detailed description of the directed evolution experiment can be found in [1].
[e] In rounds 19-22, variants were screened for a reduction in paraoxon hydrolysis and maintenance of 2NH hydrolysis. A detailed description of the directed evolution experiment can be found in [1, 2].
[f] Cells were grown in at least duplicate and lysates sufficiently diluted (~1-10,000-fold) to determine initial rates v_0 of paraoxon and 2NH hydrolysis at a substrate concentration of 200 μM, normalized to cell density, and corrected for the dilution factor. This experiment was repeated twice and the average

change of each variant relative to its respective parent (dimensionless ratio of the v0; variants used to determine the ratio are shown in the column "Calculation") and the standard deviation were determined. A detailed description of the directed evolution experiment can be found in [1, 2].
[g] A student t-test was performed to obtain p-values. Only mutants with an average >1.3-fold difference from the respective parent mutant AND a p-value <0.05 are considered significant. The cut-off of 1.3-fold was applied because only variants that differ by at least this amount from their respective parent could reliably be identified in our screening system. Non-significant values are underlined.
[h] Note that *t*199I, *l*140M and *t*45A have a >1.3 fold effect on PTE activity but non-significant p-values.

1. Tokuriki N, Jackson CJ, Afriat-Jurnou L, Wyganowski KT, Tang R, Tawfik DS. Diminishing returns and tradeoffs constrain the laboratory optimization of an enzyme. Nature Communications. 2012;3:1257.
2. Kaltenbach M, Jackson CJ, Campbell EC, Hollfelder F, Tokuriki N. Reverse evolution leads to genotypic incompatibility despite functional and active-site convergence. Elife. 2015;4.
