# Supplementary material for: Functional Trade-Offs in Promiscuous Enzymes Cannot Be Explained by Intrinsic Mutational Robustness of the Native Activity
Source: PLoS Genet. 2016 Oct 7;12(10):e1006305. doi: 10.1371/journal.pgen.1006305 (PMC5065130; doi:10.1371/journal.pgen.1006305)
Supplement: S5 Table — (PDF) [file pgen.1006305.s005.pdf]

Functional trade-offs in promiscuous enzymes cannot be explained by intrinsic mutational robustness of the native activity

S5 Table. Comparison of the effect of mutations in the evolution and in *wt*PTE. Fold-changes between the two backgrounds as well as p-values calculated according to the t-test are given.

		Paraoxon		2NH	
**Mutation[a]**	**Round[b]**	**evolution/ *wt*PTE[f]**	**T-test[f]** p-value	**evolution/ *wt*PTE[f]**	**T-test[f]** p-value
*h*254R	1	0.9	0.51	2.9	0.01
*d*233E	2/4	9.5	2.0×10^{-7}	13	7.5×10^{-9}
*f*306I	2-8[c]	/	/	/	/
*i*274S	3/4	1.2	0.32	1.0	0.87
*t*172I	5/6	7.6	8.5×10^{-5}	29	5.8×10^{-4}
*s*269T	5/6	2.0	2.0×10^{-4}	1.3	0.10
*m*138I	7/8	0.5	0.02	0.3	8.1×10^{-7}
*t*199I	7/8	1.5[g]	0.14	20	4.3×10^{-4}
*l*272M	9	0.8	0.13	1.4	0.03
*a*80V	10	1.5[g]	0.05	1.6	0.01
*s*111R	11/12	1.2	0.38	1.7	6.7×10^{-3}
*a*204G	11/12	1.7	4.3×10^{-3}	1.5	1.6×10^{-3}
*l*130V	13/14	1.5	0.01	1.6	1.8×10^{-3}
*l*271F	13/14	45	5.6×10^{-11}	3.2	1.1×10^{-6}
*a*49V	18[d]	1.2	0.03	1.5[g]	0.07
*k*77E	18[d]	1.5	8.6×10^{-3}	1.5	0.01
*l*140M	18[d]	0.8	0.21	1.0	0.71
*i*313F	18[d]	0.1	2.5×10^{-4}	1.3	0.07
*s*137T	19/20[e]	0.9	0.51	1.1	0.68
*q*180H	19/20[e]	0.3	5.0×10^{-3}	1.1	0.48
*t*45A	19/20[e]	1.6	2.6×10^{-3}	1.6	0.01
*e*144V	19/20[e]	1.5	0.03	1.6[g]	0.05
*m*314T	19/20[e]	2.1	4.4×10^{-4}	0.9	0.57
*i*341T	19/20[e]	1.0	0.86	0.9	0.39
*s*102T	21[e]	0.5	2.7×10^{-3}	1.0	0.80
*v*176M	22[e]	1.8	2.9×10^{-3}	0.9	0.31

[f] Only mutations with an average >1.3-fold difference between backgrounds AND a p-value <0.05 are considered significant. Non-significant values are underlined.
[g] Note that the effect of *t*199I and *a*80V on paraoxon hydrolysis as well as the effect of *a*49V and *e*144V on 2NH hydrolysis are statistically not significantly different between the two backgrounds.
