# Supplementary material for: Functional Trade-Offs in Promiscuous Enzymes Cannot Be Explained by Intrinsic Mutational Robustness of the Native Activity
Source: PLoS Genet. 2016 Oct 7;12(10):e1006305. doi: 10.1371/journal.pgen.1006305 (PMC5065130; doi:10.1371/journal.pgen.1006305)
Supplement: S6 Table — (PDF) [file pgen.1006305.s006.pdf]

Functional trade-offs in promiscuous enzymes cannot be explained by intrinsic mutational robustness of the native activity

S6 Table. Functional analysis of a random library of *wt*PTE variants. Changes in phosphotriesterase (PTE; native substrate: paraoxon) and arylesterase (AE; promiscuous substrate: 2NH) activities are determined relative to those of *wt*PTE by comparing the initial rates in cell lysates measured under identical conditions with 200 μM of the respective substrates (see Methods). Data (also plotted on Fig. 5A) are averages of triplicate values from three independent experiments and error values represent +/- 1 SEM.

	relative activity (vs *wt*PTE)[a]		
**Variant**	**PTE**	**AE**	**Mutation**
**1**	0.9 ± 0.2	1.0 ± 0.3	D109C
**2**	1.1 ± 0.1	1.1 ± 0.2	S47Y
**3**	< 0.01	< 0.01	n.d.
**4**	0.05 ± 0.01	< 0.01	I250M/G251L
**5**	0.03 ± 0.01	< 0.01	L262Q
**6**	< 0.01	< 0.01	Truncated (-2 bp deletion)
**7**	0.6 ± 0.04	1.2 ± 0.4	n.d.
**8**	0.6 ± 0.02	0.6 ± 0.2	n.d.
**9**	0.1 ± 0.02	0.04 ± 0.03	n.d.
**10**	1.1 ± 0.2	1.0 ± 0.1	Q343L
**11**	0.1 ± 0.03	0.2 ± 0.1	F72S/F73L
**12**	0.1 ± 0.03	0.1 ± 0.1	S75R/R76F
**13**	0.02 ± 0.01	0.02 ± 0.01	G50P
**14**	< 0.01	0.1 ± 0.1	Truncated (-1 bp deletion)
**15**	0.05 ± 0.01	< 0.01	P256STOP
**16**	0.1 ± 0.02	0.1 ± 0.1	G229N
**17**	0.6 ± 0.1	1.1 ± 0.1	n.d.
**18**	< 0.01	< 0.01	n.d.
**19**	0.9 ± 0.1	0.8 ± 0.2	R363G
**20**	0.5 ± 0.1	0.5 ± 0.2	n.d.
**21**	0.3 ± 0.03	0.1 ± 0.04	G209C
**22**	< 0.01	0.03 ± 0.03	P70R/E71STOP
**23**	0.7 ± 0.1	0.8 ± 0.03	n.d.
**24**	< 0.01	0.04 ± 0.03	L336P
**25**	< 0.01	< 0.01	n.d.
**26**	0.7 ± 0.1	1.0 ± 0.3	n.d.
**27**	0.01 ± 0.003	0.1 ± 0.1	E153A/I154L
**28**	< 0.01	0.3 ± 0.1	S307L/S308G
**29**	< 0.01	0.03 ± 0.03	Truncated (-4 bp deletion)
**30**	0.8 ± 0.1	0.9 ± 0.2	Q155L

Variant	relative activity (vs *wt*PTE)[a]		Mutation
	PTE	AE	
**31**	0.1 ± 0.02	0.5 ± 0.2	L330P
**32**	0.8 ± 0.1	0.7 ± 0.2	Y292G
**33**	1.0 ± 0.1	1.0 ± 0.1	E263Q
**34**	0.7 ± 0.1	0.8 ± 0.3	n.d.
**35**	0.2 ± 0.1	0.2 ± 0.1	T199V
**36**	0.7 ± 0.1	0.8 ± 0.2	n.d.
**37**	0.2 ± 0.04	0.3 ± 0.1	V183E
**38**	< 0.01	< 0.01	W69A
**39**	1.2 ± 0.1	0.9 ± 0.2	R67G
**40**	0.9 ± 0.01	0.9 ± 0.2	wtPTE
**41**	0.8 ± 0.05	0.9 ± 0.1	n.d.
**42**	0.9 ± 0.1	0.7 ± 0.2	wtPTE
**43**	< 0.01	< 0.01	n.d.
**44**	< 0.01	< 0.01	n.d.
**45**	0.5 ± 0.02	0.6 ± 0.2	n.d.
**46**	0.1 ± 0.02	0.4 ± 0.2	S276F/W277R
**47**	0.1 ± 0.02	< 0.01	Y248T
**48**	0.3 ± 0.02	0.9 ± 0.2	n.d.
**49**	< 0.01	0.1 ± 0.1	S62F/A63T
**50**	0.8 ± 0.0	1.2 ± 0.2	n.d.
**51**	< 0.01	0.2 ± 0.2	n.d.
**52**	1.0 ± 0.3	0.8 ± 0.1	L282S
**53**	< 0.01	< 0.01	n.d.
**54**	0.2 ± 0.03	0.1 ± 0.1	G229A
**55**	1.0 ± 0.1	0.9 ± 0.1	Q343G
**56**	< 0.01	< 0.01	G60C
**57**	0.2 ± 0.04	0.1 ± 0.04	L151D
**58**	< 0.01	< 0.01	E48STOP
**59**	0.7 ± 0.1	0.6 ± 0.2	n.d.
**60**	0.6 ± 0.05	0.5 ± 0.2	n.d.
**61**	0.7 ± 0.1	0.7 ± 0.3	n.d.
**62**	1.0 ± 0.2	0.8 ± 0.2	A93V
**63**	< 0.01	< 0.01	V143A/E144STOP
**64**	0.02 ± 0.005	0.9 ± 0.3	n.d.
**65**	0.9 ± 0.05	0.7 ± 0.03	A92G/A93T
**66**	< 0.01	< 0.01	n.d.
**67**	< 0.01	< 0.01	D100Y
**68**	0.8 ± 0.1	0.7 ± 0.1	R96T
**69**	0.9 ± 0.2	0.8 ± 0.1	S102G
**70**	0.2 ± 0.1	0.8 ± 0.2	n.d.
**71**	1.0 ± 0.1	0.9 ± 0.1	R189 silent
**72**	0.8 ± 0.1	0.7 ± 0.1	n.d.
**73**	0.4 ± 0.2	0.9 ± 0.4	n.d.
**74**	0.9 ± 0.04	0.7 ± 0.1	n.d.

Variant	relative activity (vs *wt*PTE)[a]		Mutation
	PTE	AE	
**75**	< 0.01	< 0.01	n.d.
**76**	0.2 ± 0.1	0.5 ± 0.4	n.d.
**77**	0.4 ± 0.1	0.9 ± 0.2	n.d.
**78**	1.0 ± 0.2	1.0 ± 0.1	V143A
**79**	< 0.01	< 0.01	n.d.
**80**	0.9 ± 0.1	1.5 ± 0.2	A266D
**81**	0.6 ± 0.05	0.5 ± 0.1	n.d.
**82**	0.8 ± 0.01	0.7 ± 0.1	R118K
**83**	0.8 ± 0.1	0.7 ± 0.1	n.d.
**84**	0.3 ± 0.1	0.3 ± 0.2	L336A
**85**	0.1 ± 0.01	2.0 ± 0.3	L271T
**86**	< 0.01	< 0.01	n.d.
**87**	1.0 ± 0.4	1.0 ± 0.2	n.d.
**88**	1.1 ± 0.2	1.3 ± 0.1	E144Q
**89**	0.7 ± 0.1	0.7 ± 0.1	n.d.
**90**	0.9 ± 0.2	1.0 ± 0.1	E144I
**91**	< 0.01	0.02 ± 0.02	n.d.
**92**	0.8 ± 0.1	0.8 ± 0.1	n.d.
**93**	< 0.01	0.02 ± 0.01	n.d.
**94**	0.5 ± 0.05	0.6 ± 0.1	n.d.
**95**	0.6 ± 0.02	0.5 ± 0.05	n.d.
**96**	< 0.01	0.02 ± 0.02	n.d.
**97**	< 0.01	0.05 ± 0.05	n.d.
**98**	1.0 ± 0.2	0.9 ± 0.1	A80G
**99**	0.03 ± 0.002	1.2 ± 0.2	n.d.
**100**	0.6 ± 0.1	0.7 ± 0.1	n.d.
**101**	0.1 ± 0.01	0.1 ± 0.04	n.d.
**102**	< 0.01	0.2 ± 0.1	n.d.
**103**	< 0.01	0.02 ± 0.02	n.d.
**104**	< 0.01	0.1 ± 0.04	n.d.
**105**	< 0.01	0.1 ± 0.03	n.d.
**106**	0.7 ± 0.1	0.3 ± 0.1	n.d.
**107**	1.0 ± 0.2	3.1 ± 0.3	F73C
**108**	0.04 ± 0.04	0.1 ± 0.1	n.d.
**109**	< 0.01	0.02 ± 0.02	n.d.
**110**	< 0.01	0.02 ± 0.02	n.d.
**111**	0.02 ± 0.003	0.04 ± 0.04	n.d.
**112**	< 0.01	0.04 ± 0.02	n.d.
**113**	0.4 ± 0.1	0.6 ± 0.1	n.d.
**114**	< 0.01	0.02 ± 0.01	n.d.
**115**	0.1 ± 0.1	0.02 ± 0.02	n.d.
**116**	< 0.01	< 0.01	n.d.
**117**	1.1 ± 0.1	1.1 ± 0.1	R118G
**118**	0.4 ± 0.02	0.4 ± 0.1	n.d.

Variant	relative activity (vs *wt*PTE)[a]		Mutation
	PTE	AE	
**119**	0.5 ± 0.01	0.4 ± 0.1	n.d.
**120**	0.1 ± 0.03	2.0 ± 0.04	L272R/G273R
**121**	0.6 ± 0.04	0.5 ± 0.1	n.d.
**122**	0.9 ± 0.1	1.1 ± 0.01	R331G
**123**	< 0.01	0.03 ± 0.02	n.d.
**124**	0.1 ± 0.03	0.1 ± 0.1	n.d.
**125**	1.0 ± 0.1	1.0 ± 0.1	N312D
**126**	< 0.01	0.04 ± 0.04	n.d.
**127**	0.8 ± 0.1	0.7 ± 0.1	n.d.
**128**	< 0.01	0.04 ± 0.04	n.d.
**129**	< 0.01	0.03 ± 0.02	n.d.
**130**	0.1 ± 0.01	0.1 ± 0.05	n.d.
**131**	< 0.01	0.03 ± 0.01	n.d.
**132**	0.7 ± 0.1	0.5 ± 0.1	n.d.
**133**	1.1 ± 0.1	1.0 ± 0.1	L330S
**134**	< 0.01	0.04 ± 0.04	n.d.
**135**	0.2 ± 0.1	0.2 ± 0.1	n.d.
**136**	1.0 ± 0.1	1.0 ± 0.05	M293T
**137**	1.0 ± 0.1	1.0 ± 0.02	R118G
**138**	0.9 ± 0.1	1.2 ± 0.02	S75G
**139**	0.9 ± 0.02	0.8 ± 0.1	T147S
**140**	0.2 ± 0.1	0.2 ± 0.1	n.d.
**141**	< 0.01	0.02 ± 0.02	n.d.
**142**	1.1 ± 0.2	1.0 ± 0.1	L330I
**143**	< 0.01	0.02 ± 0.02	n.d.
**144**	< 0.01	0.02 ± 0.02	n.d.
**145**	< 0.01	0.02 ± 0.02	n.d.
**146**	0.8 ± 0.03	0.7 ± 0.1	n.d.
**147**	1.1 ± 0.02	1.0 ± 0.1	P342L/Q343E
**148**	0.3 ± 0.01	0.2 ± 0.1	n.d.
**149**	0.6 ± 0.04	0.5 ± 0.1	n.d.
**150**	0.8 ± 0.1	0.9 ± 0.1	n.d.
**151**	0.9 ± 0.1	0.8 ± 0.1	n.d.
**152**	< 0.01	0.1 ± 0.03	n.d.
**153**	0.9 ± 0.03	1.3 ± 0.03	L262H
**154**	1.1 ± 0.1	1.0 ± 0.03	Q343A
**155**	0.1 ± 0.005	2.3 ± 0.3	L272H/G273R
**156**	0.6 ± 0.1	0.7 ± 0.1	n.d.
**157**	0.9 ± 0.1	0.8 ± 0.1	I44K/T45A
**158**	0.4 ± 0.02	0.5 ± 0.1	n.d.
**159**	< 0.01	0.04 ± 0.04	n.d.
**160**	1.2 ± 0.1	1.1 ± 0.1	S47Y
**161**	0.2 ± 0.1	0.3 ± 0.1	n.d.
**162**	0.1 ± 0.01	0.1 ± 0.04	n.d.

Variant	relative activity (vs *wt*PTE)[a]		Mutation
	PTE	AE	
**163**	0.7 ± 0.1	0.8 ± 0.1	n.d.
**164**	< 0.01	2.1 ± 0.4	I313K
**165**	< 0.01	0.02 ± 0.02	n.d.
**166**	1.1 ± 0.2	1.0 ± 0.1	S117 silent
**167**	0.1 ± 0.04	0.1 ± 0.1	n.d.
**168**	0.03 ± 0.01	2.9 ± 0.6	M314K/D315Y
**169**	0.03 ± 0.002	0.1 ± 0.03	n.d.
**170**	1.0 ± 0.1	0.9 ± 0.1	n.d.
**171**	1.0 ± 0.1	1.3 ± 0.1	n.d.
**172**	1.1 ± 0.1	1.0 ± 0.1	Y292R
**173**	1.0 ± 0.2	0.8 ± 0.1	D323E/G324R
**174**	1.0 ± 0.2	2.4 ± 0.7	T54S
**175**	1.2 ± 0.1	1.0 ± 0.1	Y292F
**176**	0.7 ± 0.1	0.5 ± 0.1	n.d.
**177**	0.9 ± 0.1	0.7 ± 0.2	L330A
**178**	< 0.01	< 0.01	n.d.
**179**	< 0.01	0.02 ± 0.02	n.d.
**180**	< 0.01	< 0.01	n.d.
**181**	0.7 ± 0.04	0.9 ± 0.1	n.d.
**182**	0.8 ± 0.04	0.7 ± 0.1	L262M
**183**	0.9 ± 0.1	0.6 ± 0.1	G348 silent
**184**	0.1 ± 0.03	0.1 ± 0.1	n.d.
**185**	0.8 ± 0.1	0.7 ± 0.1	A242P
**186**	0.7 ± 0.1	1.1 ± 0.4	n.d.
**187**	0.6 ± 0.1	0.8 ± 0.2	n.d.
**188**	0.7 ± 0.02	0.5 ± 0.1	n.d.
**189**	1.0 ± 0.1	0.5 ± 0.1	D109S
**190**	< 0.01	0.02 ± 0.02	n.d.
**191**	0.9 ± 0.1	1.3 ± 0.1	R89F
**192**	0.1 ± 0.01	0.03 ± 0.02	n.d.
**193**	0.01 ± 0.01	< 0.01	n.d.
**194**	0.8 ± 0.1	0.5 ± 0.1	n.d.
**195**	0.9 ± 0.1	0.6 ± 0.1	P342L
**196**	0.1 ± 0.01	1.4 ± 0.4	L272Q/G273S
**197**	< 0.01	< 0.01	n.d.
**198**	< 0.01	0.01 ± 0.01	n.d.
**199**	0.7 ± 0.03	0.7 ± 0.03	n.d.
**200**	0.7 ± 0.1	0.6 ± 0.1	n.d.
**201**	0.3 ± 0.1	3.4 ± 1.3	K339I/G340R
**202**	0.02 ± 0.004	< 0.01	n.d.
**203**	0.6 ± 0.04	0.6 ± 0.2	n.d.
**204**	< 0.01	< 0.01	n.d.
**205**	< 0.01	< 0.01	n.d.
**206**	< 0.01	< 0.01	n.d.

Variant	relative activity (vs *wt*PTE)[a]		Mutation
	PTE	AE	
**207**	< 0.01	0.02 ± 0.02	n.d.
**208**	0.1 ± 0.01	0.01 ± 0.01	n.d.
**209**	< 0.01	< 0.01	n.d.
**210**	< 0.01	< 0.01	n.d.
**211**	0.5 ± 0.1	0.6 ± 0.1	n.d.
**212**	0.4 ± 0.04	0.3 ± 0.1	n.d.
**213**	0.9 ± 0.1	0.7 ± 0.1	L151R
**214**	< 0.01	< 0.01	n.d.
**215**	< 0.01	0.02 ± 0.02	n.d.
**216**	0.2 ± 0.01	1.0 ± 0.2	n.d.
**217**	0.6 ± 0.1	1.2 ± 0.1	n.d.
**218**	1.1 ± 0.1	0.6 ± 0.1	A80G/E81K
**219**	1.0 ± 0.1	0.6 ± 0.2	wtPTE
**220**	0.03 ± 0.01	0.7 ± 0.4	n.d.
**221**	0.3 ± 0.01	0.1 ± 0.1	n.d.
**222**	0.2 ± 0.04	0.1 ± 0.05	n.d.
**223**	0.8 ± 0.1	0.8 ± 0.1	n.d.
**224**	0.3 ± 0.1	0.4 ± 0.1	n.d.
**225**	1.0 ± 0.2	0.7 ± 0.1	wtPTE
**226**	0.3 ± 0.03	0.1 ± 0.1	n.d.
**227**	1.1 ± 0.1	0.6 ± 0.2	n.d.
**228**	< 0.01	< 0.01	n.d.
**229**	0.9 ± 0.04	0.6 ± 0.2	P334N
**230**	1.1 ± 0.1	1.0 ± 0.1	P70 silent
**231**	< 0.01	0.03 ± 0.03	n.d.
**232**	0.03 ± 0.004	1.7 ± 0.02	M317S
**233**	0.7 ± 0.1	0.5 ± 0.2	n.d.
**234**	< 0.01	< 0.01	n.d.
**235**	0.4 ± 0.1	0.2 ± 0.1	n.d.
**236**	0.4 ± 0.1	0.3 ± 0.3	n.d.
**237**	0.2 ± 0.04	0.1 ± 0.1	n.d.
**238**	< 0.01	< 0.01	n.d.
**239**	0.01 ± 0.001	0.5 ± 0.1	n.d.
**240**	0.8 ± 0.04	0.8 ± 0.02	G162R
**241**	1.1 ± 0.3	0.8 ± 0.1	R356E
**242**	0.3 ± 0.03	0.6 ± 0.3	n.d.
**243**	0.8 ± 0.2	2.0 ± 0.5	K339T
**244**	1.2 ± 0.2	0.6 ± 0.1	R118Q
**245**	< 0.01	0.03 ± 0.03	n.d.
**246**	0.02 ± 0.01	1.2 ± 0.2	n.d.
**247**	0.6 ± 0.1	0.4 ± 0.03	n.d.
**248**	1.1 ± 0.1	1.0 ± 0.04	R36N
**249**	< 0.01	< 0.01	n.d.
**250**	1.0 ± 0.2	0.5 ± 0.1	G162V

Variant	relative activity (vs *wt*PTE)[a]		Mutation
	PTE	AE	
**251**	< 0.01	< 0.01	n.d.
**252**	1.0 ± 0.2	0.5 ± 0.1	A63T
**253**	0.7 ± 0.2	0.3 ± 0.1	n.d.
**254**	0.04 ± 0.01	0.6 ± 0.1	n.d.
**255**	1.2 ± 0.1	1.1 ± 0.05	S47C/E48Q
**256**	0.4 ± 0.03	0.2 ± 0.04	n.d.
**257**	0.04 ± 0.002	0.3 ± 0.1	n.d.
**258**	0.7 ± 0.1	0.5 ± 0.1	n.d.
**259**	0.03 ± 0.01	< 0.01	n.d.
**260**	1.1 ± 0.2	0.7 ± 0.1	R337Y
**261**	1.3 ± 0.5	0.7 ± 0.1	n.d.
**262**	0.2 ± 0.1	0.1 ± 0.1	n.d.
**263**	1.0 ± 0.04	1.0 ± 0.3	L262 silent
**264**	0.5 ± 0.1	1.6 ± 0.5	T311A
**265**	< 0.01	< 0.01	n.d.
**266**	1.0 ± 0.01	1.2 ± 0.3	Q206L
**267**	0.6 ± 0.1	0.5 ± 0.1	n.d.
**268**	< 0.01	1.3 ± 0.5	Y309R
**269**	0.6 ± 0.1	0.6 ± 0.2	n.d.
**270**	1.0 ± 0.2	0.9 ± 0.3	wtPTE
**271**	1.1 ± 0.2	0.8 ± 0.2	V351D
**272**	0.9 ± 0.1	1.0 ± 0.2	L182 silent
**273**	< 0.01	0.1 ± 0.1	n.d.
**274**	0.03 ± 0.0003	0.01 ± 0.01	n.d.
**275**	< 0.01	0.02 ± 0.02	n.d.
**276**	1.1 ± 0.1	1.2 ± 0.4	S238G
**277**	0.9 ± 0.1	1.2 ± 0.05	A63S
**278**	0.9 ± 0.1	0.9 ± 0.1	L262I
**279**	< 0.01	< 0.01	n.d.
**280**	0.9 ± 0.1	0.8 ± 0.1	V198L
**281**	0.7 ± 0.1	0.8 ± 0.1	n.d.
**282**	0.4 ± 0.1	0.3 ± 0.2	n.d.
**283**	< 0.01	0.1 ± 0.1	n.d.
**284**	< 0.01	0.2 ± 0.1	n.d.
**285**	1.0 ± 0.1	1.3 ± 0.2	L262 silent
**286**	< 0.01	0.05 ± 0.05	n.d.
**287**	0.04 ± 0.01	0.5 ± 0.2	n.d.
**288**	< 0.01	< 0.01	n.d.
**289**	0.5 ± 0.1	0.9 ± 0.2	n.d.
**290**	0.4 ± 0.02	2.8 ± 0.4	n.d.
**291**	0.1 ± 0.02	0.04 ± 0.04	n.d.
**292**	1.1 ± 0.2	1.0 ± 0.3	S102T
**293**	< 0.01	0.1 ± 0.1	n.d.
**294**	0.04 ± 0.01	0.1 ± 0.1	n.d.

Variant	relative activity (vs *wt*PTE)[a]		Mutation
	PTE	AE	
**295**	0.4 ± 0.2	1.1 ± 0.2	n.d.
**296**	< 0.01	0.02 ± 0.02	n.d.
**297**	0.5 ± 0.1	0.6 ± 0.2	n.d.
**298**	0.5 ± 0.2	1.0 ± 0.1	n.d.
**299**	0.5 ± 0.1	1.8 ± 0.3	L262E
**300**	0.9 ± 0.3	0.9 ± 0.2	R67T
**301**	< 0.01	0.01 ± 0.01	n.d.
**302**	< 0.01	< 0.01	n.d.
**303**	0.2 ± 0.1	0.9 ± 0.4	n.d.
**304**	< 0.01	< 0.01	n.d.
**305**	0.1 ± 0.01	4.4 ± 2.3	R280S
**306**	0.2 ± 0.04	0.3 ± 0.3	n.d.
**307**	0.7 ± 0.1	0.8 ± 0.3	n.d.
**308**	< 0.01	0.03 ± 0.03	n.d.
**309**	0.7 ± 0.1	0.7 ± 0.1	n.d.
**310**	< 0.01	0.04 ± 0.04	n.d.
**311**	0.8 ± 0.2	0.7 ± 0.2	Q206H
**312**	0.5 ± 0.1	0.5 ± 0.2	n.d.
**313**	0.9 ± 0.1	0.7 ± 0.2	P134 silent
**314**	0.01 ± 0.002	< 0.01	n.d.
**315**	0.02 ± 0.002	0.01 ± 0.01	n.d.
**316**	< 0.01	0.03 ± 0.03	n.d.
**317**	< 0.01	< 0.01	n.d.
**318**	0.8 ± 0.1	0.8 ± 0.2	S365R
**319**	0.8 ± 0.2	0.5 ± 0.2	D100A
**320**	0.9 ± 0.2	0.7 ± 0.2	G208A
**321**	< 0.01	< 0.01	n.d.
**322**	0.9 ± 0.2	0.6 ± 0.3	Y292R
**323**	< 0.01	0.02 ± 0.02	n.d.
**324**	0.1 ± 0.03	0.1 ± 0.1	n.d.
**325**	0.3 ± 0.1	0.2 ± 0.2	n.d.
**326**	< 0.01	0.1 ± 0.1	n.d.
**327**	0.9 ± 0.1	0.7 ± 0.2	Q343I
**328**	0.8 ± 0.1	0.7 ± 0.2	I44T/T45A
**329**	0.6 ± 0.1	0.5 ± 0.2	n.d.
**330**	< 0.01	< 0.01	n.d.
**331**	0.3 ± 0.1	0.5 ± 0.4	n.d.
**332**	0.1 ± 0.1	0.1 ± 0.1	n.d.
**333**	< 0.01	0.03 ± 0.03	n.d.
**334**	0.6 ± 0.1	0.5 ± 0.2	n.d.
**335**	0.9 ± 0.3	1.0 ± 0.3	P342W
**336**	0.8 ± 0.2	0.7 ± 0.2	K339R
**337**	0.1 ± 0.02	0.1 ± 0.1	n.d.
**338**	0.5 ± 0.1	1.9 ± 0.7	n.d.

Variant	relative activity (vs *wt*PTE)[a] PTE	AE	Mutation
**339**	0.05 ± 0.003	0.04 ± 0.04	n.d.
**340**	0.7 ± 0.04	0.6 ± 0.2	n.d.
**341**	0.3 ± 0.03	0.2 ± 0.1	n.d.
**342**	0.2 ± 0.1	0.7 ± 0.2	n.d.
**343**	1.0 ± 0.2	0.5 ± 0.3	Y292F
**344**	0.2 ± 0.05	0.2 ± 0.2	n.d.
**345**	0.9 ± 0.1	0.7 ± 0.2	L221 silent
**346**	0.4 ± 0.1	3.9 ± 1.9	K339T/G340C
**347**	< 0.01	0.02 ± 0.02	n.d.
**348**	< 0.01	< 0.01	n.d.
**349**	< 0.01	1.3 ± 0.2	n.d.
**350**	0.1 ± 0.004	6.6 ± 2.2	R280T
**351**	1.2 ± 0.04	3.1 ± 0.8	S269H
**352**	< 0.01	3.5 ± 1.1	ΔM317
**353**	< 0.01	0.04 ± 0.04	n.d.
**354**	< 0.01	0.4 ± 0.1	n.d.
**355**	0.1 ± 0.03	0.2 ± 0.1	n.d.
**356**	0.8 ± 0.1	1.0 ± 0.1	Q343W
**357**	< 0.01	0.03 ± 0.02	n.d.
**358**	1.1 ± 0.2	1.2 ± 0.2	E263Q
**359**	1.2 ± 0.2	1.1 ± 0.1	Y292L
**360**	1.0 ± 0.2	1.1 ± 0.1	V143 silent
**361**	< 0.01	0.02 ± 0.02	n.d.
**362**	< 0.01	< 0.01	n.d.
**363**	1.1 ± 0.2	1.4 ± 0.3	Q343L
**364**	1.0 ± 0.04	1.0 ± 0.1	P342R
**365**	1.0 ± 0.1	0.9 ± 0.1	A364 silent
**366**	0.4 ± 0.03	0.2 ± 0.1	n.d.
**367**	0.1 ± 0.1	0.1 ± 0.1	n.d.
**368**	1.1 ± 0.3	1.0 ± 0.1	T241I
**369**	0.2 ± 0.03	0.7 ± 0.2	n.d.
**370**	0.4 ± 0.1	0.7 ± 0.03	n.d.
**371**	0.2 ± 0.01	3.3 ± 0.8	V310A/ΔT311
**372**	0.7 ± 0.1	0.6 ± 0.1	n.d.
**373**	0.5 ± 0.1	0.5 ± 0.1	n.d.
**374**	0.8 ± 0.1	0.9 ± 0.01	E219 silent
**375**	0.2 ± 0.04	0.03 ± 0.03	n.d.
**376**	< 0.01	2.3 ± 0.4	H254P
**377**	< 0.01	0.02 ± 0.02	n.d.
**378**	< 0.01	0.03 ± 0.03	n.d.
**379**	< 0.01	0.04 ± 0.03	n.d.
**380**	0.7 ± 0.1	1.1 ± 0.1	n.d.
**381**	0.9 ± 0.02	1.1 ± 0.02	P334T
**382**	0.02 ± 0.02	0.01 ± 0.01	n.d.

Variant	relative activity (vs *wt*PTE)[a]		Mutation
	PTE	AE	
**383**	0.3 ± 0.1	0.3 ± 0.1	n.d.
**384**	< 0.01	0.1 ± 0.1	n.d.
**385**	0.2 ± 0.01	1.9 ± 0.3	L272C
**386**	0.8 ± 0.1	9.1 ± 3.9	W302F
**387**	0.04 ± 0.004	0.1 ± 0.03	n.d.
**388**	1.1 ± 0.1	1.0 ± 0.1	Q343P
**389**	< 0.01	< 0.01	n.d.
**390**	< 0.01	0.01 ± 0.01	n.d.
**391**	0.1 ± 0.01	2.2 ± 0.2	L271A
**392**	< 0.01	< 0.01	n.d.
**393**	1.1 ± 0.2	1.1 ± 0.1	P223A
**394**	1.2 ± 0.2	1.3 ± 0.2	n.d.
**395**	0.6 ± 0.1	0.7 ± 0.1	n.d.
**396**	< 0.01	0.01 ± 0.01	n.d.
**397**	1.0 ± 0.1	1.0 ± 0.03	P329 silent
**398**	0.4 ± 0.03	0.4 ± 0.1	n.d.
**399**	< 0.01	< 0.01	n.d.
**400**	0.6 ± 0.1	0.6 ± 0.1	n.d.
**401**	0.9 ± 0.1	1.2 ± 0.1	P334A
**402**	< 0.01	< 0.01	n.d.
**403**	1.1 ± 0.3	1.1 ± 0.1	n.d.
**404**	< 0.01	0.02 ± 0.02	n.d.
**405**	< 0.01	0.04 ± 0.02	n.d.
**406**	0.2 ± 0.1	0.1 ± 0.1	n.d.
**407**	0.4 ± 0.1	0.7 ± 0.2	n.d.
**408**	0.1 ± 0.01	2.6 ± 0.5	L272P
**409**	< 0.01	0.04 ± 0.04	n.d.
**410**	1.5 ± 0.3	1.3 ± 0.2	Q343E
**411**	0.02 ± 0.01	0.1 ± 0.1	n.d.
**412**	0.9 ± 0.1	2.0 ± 0.2	D235T
**413**	< 0.01	0.03 ± 0.02	n.d.
**414**	0.8 ± 0.04	0.8 ± 0.1	n.d.
**415**	1.3 ± 0.4	0.4 ± 0.2	S308C
**416**	1.0 ± 0.2	0.7 ± 0.1	S269A
**417**	< 0.01	0.04 ± 0.04	n.d.
**418**	< 0.01	0.02 ± 0.02	n.d.
**419**	< 0.01	0.03 ± 0.03	n.d.
**420**	1.0 ± 0.2	1.4 ± 0.2	A266G
**421**	0.01 ± 0.01	0.03 ± 0.03	n.d.
**422**	< 0.01	0.02 ± 0.02	n.d.
**423**	1.1 ± 0.2	1.0 ± 0.1	A49M
**424**	0.7 ± 0.2	0.6 ± 0.1	n.d.
**425**	1.1 ± 0.2	1.2 ± 0.2	G348A
**426**	< 0.01	< 0.01	n.d.

Variant	relative activity (vs *wt*PTE)[a]		Mutation
	**PTE**	**AE**	
**427**	1.3 ± 0.2	1.3 ± 0.2	T117N/P118A
**428**	< 0.01	0.03 ± 0.03	n.d.
**429**	1.3 ± 0.2	1.0 ± 0.1	n.d.
**430**	0.4 ± 0.1	0.3 ± 0.1	n.d.
**431**	< 0.01	0.1 ± 0.05	n.d.
**432**	0.8 ± 0.2	1.0 ± 0.2	V143A/E144K
**433**	1.2 ± 0.1	1.2 ± 0.1	R67H
**434**	0.01 ± 0.01	< 0.01	n.d.
**435**	1.5 ± 0.2	1.5 ± 0.3	n.d.

[a] After cell growth, protein expression and lysis, lysates were sufficiently diluted (~1-10,000-fold) to determine initial rates v_0 of paraoxon and 2NH hydrolysis at a substrate concentration of 200 μM, normalized to cell density, and corrected for the dilution factor. This experiment was performed three times and the average change relative to *wt*PTE and the standard error of the mean (SEM) were determined. The relative activity shown represents the dimensionless ratio of the v_o of each variant compared to that of *wt*PTE.
