# Supplementary material for: Functional Trade-Offs in Promiscuous Enzymes Cannot Be Explained by Intrinsic Mutational Robustness of the Native Activity
Source: PLoS Genet. 2016 Oct 7;12(10):e1006305. doi: 10.1371/journal.pgen.1006305 (PMC5065130; doi:10.1371/journal.pgen.1006305)
Supplement: S7 Table — (PDF) [file pgen.1006305.s007.pdf]

# Functional trade-offs in promiscuous enzymes cannot be explained by intrinsic mutational robustness of the native activity

**S7 Table. Statistical parameters derived from the functional characterization of the random library of *wt*PTE variants.** The difference between the distributions of mutational changes for PTE and AE activities is statistically significant (Two-sample Kolmogorov-Smirnov test, $p = 1.8 \times 10^{-5}$).

| | | PTE | AE |
|---|---|---|---|
| **All variants** (n = 435) | *Average mutational change*[a][b] | 0.14 [0.11 ; 0.17] | 0.21 [0.18 ; 0.25] |
| | *Median mutational change*[a] | 0.31 | 0.53 |
| | *Maximum mutational change*[a] | 1.50 | 9.11 |
| | *Minimum mutational change*[a] | < 0.01 | < 0.01 |
| **AE positive variants** (n = 34) | *Average mutational change*[a][b] | 0.21 [0.12 ; 0.36] | 2.30 [1.97 ; 2.68] |
| | *Median mutational change*[a][b] | 0.33 | 2.09 |
| | *Maximum mutational change*[a] | 1.50 | 9.11 |
| | *Minimum mutational change*[a] | < 0.01 | 1.30 |

[a] Values refer to the activity change of all or AE positive variants relative to *wt*PTE obtained by comparing the initial rates $v_0$ for the hydrolysis of paraoxon (PTE) or 2-NH (AE) to that of *wt*PTE at 200 µM substrate concentration, resulting in a dimensionless ratio.

[b] The average mutational change refers to the change in initial rates as a consequence of mutation and was calculated as the geometric mean of the relative activities of the variants (see Table S5). The corresponding confidence intervals (5% risk of error) are indicated between brackets.
