# Supplementary material for: Functional Trade-Offs in Promiscuous Enzymes Cannot Be Explained by Intrinsic Mutational Robustness of the Native Activity
Source: PLoS Genet. 2016 Oct 7;12(10):e1006305. doi: 10.1371/journal.pgen.1006305 (PMC5065130; doi:10.1371/journal.pgen.1006305)
Supplement: S8 Table — (PDF) [file pgen.1006305.s008.pdf]

Functional trade-offs in promiscuous enzymes cannot be explained by intrinsic mutational robustness of the native activity

S8 Table. Overview of the 9 mutations in the evolution from AtzA to TriA (adapted from reference [1]). Mutations are shown relative to AtzA with lower case italics denoting the amino acid found in AtzA.

		Mutations								
Round	Variant	*s331*	*f84*	*n328*	*e125*	*t219*	*t217*	*v92*	*g255*	*i253*
0	AtzA									
1		C								
2		C	L							
3		C	L	D						
4		C	L	D	D					
5		C	L	D	D	P				
6		C	L	D	D	P	I			
7		C	L	D	D	P	I	L		
8		C	L	D	D	P	I	L	W	
9	TriA	C	L	D	D	P	I	L	W	L

1. Noor S, Taylor MC, Russell RJ, Jermiin LS, Jackson CJ, Oakeshott JG, et al. Intramolecular epistasis and the evolution of a new enzymatic function. PLoS One. 2012;7(6):e39822.
