# Supplementary material for: Functional Trade-Offs in Promiscuous Enzymes Cannot Be Explained by Intrinsic Mutational Robustness of the Native Activity
Source: PLoS Genet. 2016 Oct 7;12(10):e1006305. doi: 10.1371/journal.pgen.1006305 (PMC5065130; doi:10.1371/journal.pgen.1006305)
Supplement: S9 Table — (PDF) [file pgen.1006305.s009.pdf]

Functional trade-offs in promiscuous enzymes cannot be explained by intrinsic mutational robustness of the native activity

S9 Table. Effect of mutations on atrazine dechlorination (adapted from reference [1]).

Mutation[a]	Round	k_{cat}/K_M $[M^{-1}s^{-1}]$[b]			relative activity[c]		
		AtzA	In the evolution	TriA	AtzA	In the evolution	TriA
/		$(1.5\pm0.1)\times10^{4}$	$(1.5\pm0.1)\times10^{4}$	70±5			
*s*331C	1	$(3.2\pm0.2)\times10^{3}$	$(3.2\pm0.2)\times10^{3}$	$(1.7\pm0.1)\times10^{3}$	0.2	0.2	24
*f*84L	2	$(2.4\pm0.1)\times10^{3}$	$(6.5\pm0.6)\times10^{2}$	$(8.2\pm0.7)\times10^{2}$	0.2	0.2	12
*n*328D	3	$(6.0\pm0.03)\times10^{2}$	$(5.1\pm0.4)\times10^{2}$	n.d.[d]	0.0	0.8	/
*e*125D	4	$(1.4\pm0.03)\times10^{4}$	$(2.1\pm0.1)\times10^{2}$	79±8	1.0	0.4	1.1
*t*219P	5	$(1.4\pm0.1)\times10^{4}$	96±7	93±4	1.0	0.5	1.3
*t*217I	6	$(1.4\pm0.1)\times10^{2}$	80±7	97±7	1.0	0.8	1.4
*v*92L	7	$(1.4\pm0.1)\times10^{3}$	55±6	73±7	1.0	0.7	1.0
g255W	8	$(1.3\pm0.1)\times10^{3}$	57±5	69±6	1.0	1.0	1.0
*i*253L	9	$(1.6\pm0.02)\times10^{3}$	60±6	82±5	1.1	1.1	1.2

[a] Amino acids present in AtzA are shown in lower-case italics.
[b] k_{cat}/K_M values were taken from reference [1].
[c] Fold-changes were calculated relative to AtzA or TriA. To determine fold-changes in the evolution, activities were calculated relative to the respective preceding variant, *i.e.* the variant lacking the mutation in question.
[d] Activity was not detected.

1. Noor S, Taylor MC, Russell RJ, Jermiin LS, Jackson CJ, Oakeshott JG, et al. Intramolecular epistasis and the evolution of a new enzymatic function. PLoS One. 2012;7(6):e39822.
