# Supplementary material for: Functional Trade-Offs in Promiscuous Enzymes Cannot Be Explained by Intrinsic Mutational Robustness of the Native Activity
Source: PLoS Genet. 2016 Oct 7;12(10):e1006305. doi: 10.1371/journal.pgen.1006305 (PMC5065130; doi:10.1371/journal.pgen.1006305)
Supplement: S10 Table — (PDF) [file pgen.1006305.s010.pdf]

Functional trade-offs in promiscuous enzymes cannot be explained by intrinsic mutational robustness of the native activity

S10 Table. Effect of mutations on melamine deamination (adapted from reference [1]).

		k_{cat}/K_M [$M^{-1}s^{-1}$][b]			relative activity[c]		
**Mutation[a]**	**Round**	**AtzA**	**In the evolution**	**TriA**	**AtzA**	**In the evolution**	**TriA**
/		n.d.[d]		(1.6±0.1)×10^4	/	/	/
*s*331C	1	50±4	50±4	(3.3±0.3)×10^3	/	/	/
*f*84L	2	n.d.[d]	(9.1±0.8)×10^2	(6.1±0.3)×10^3	/	/	/
*n*328D	3	n.d.[d]	(6.1±0.6)×10^3	n.d.[d]	/	/	/
*e*125D	4	n.d.[d]	(1.1±0.1)×10^4	(8.9±0.8)×10^3	/	/	/
*t*219P	5	n.d.[d]	(1.9±0.1)×10^4	(1.1±0.1)×10^4	/	/	/
*t*217I	6	n.d.[d]	(1.9±0.1)×10^4	(9.3±0.6)×10^3	/	/	/
*v*92L	7	n.d.[d]	(2.0±0.2)×10^4	(1.1±0.1)×10^4	/	/	/
g255W	8	n.d.[d]	(2.1±0.2)×10^4	(1.3±0.1)×10^4	/	/	/
*i*253L	9	n.d.[d]	(2.1±0.2)×10^4	(1.6±0.02)×10^4	/	/	/

[a] Amino acids present in AtzA are shown in lower-case italics.
[b] k_{cat}/K_M values were taken from reference [1].
[c] Fold-changes were calculated relative to AtzA or TriA. To determine fold-changes in the evolution, activities were calculated relative to the respective preceding variant, *i.e.* the variant lacking the mutation in question.
[d] Activity was not detected.

1. Noor S, Taylor MC, Russell RJ, Jermiin LS, Jackson CJ, Oakeshott JG, et al. Intramolecular epistasis and the evolution of a new enzymatic function. PLoS One. 2012;7(6):e39822.
