# Supplementary material for: Functional Trade-Offs in Promiscuous Enzymes Cannot Be Explained by Intrinsic Mutational Robustness of the Native Activity
Source: PLoS Genet. 2016 Oct 7;12(10):e1006305. doi: 10.1371/journal.pgen.1006305 (PMC5065130; doi:10.1371/journal.pgen.1006305)
Supplement: S1 Fig — (PDF) [file pgen.1006305.s011.pdf]

Supporting Information for Kaltenbach at al.,

Functional trade-offs in promiscuous enzymes cannot be explained by intrinsic mutational robustness of the native activity

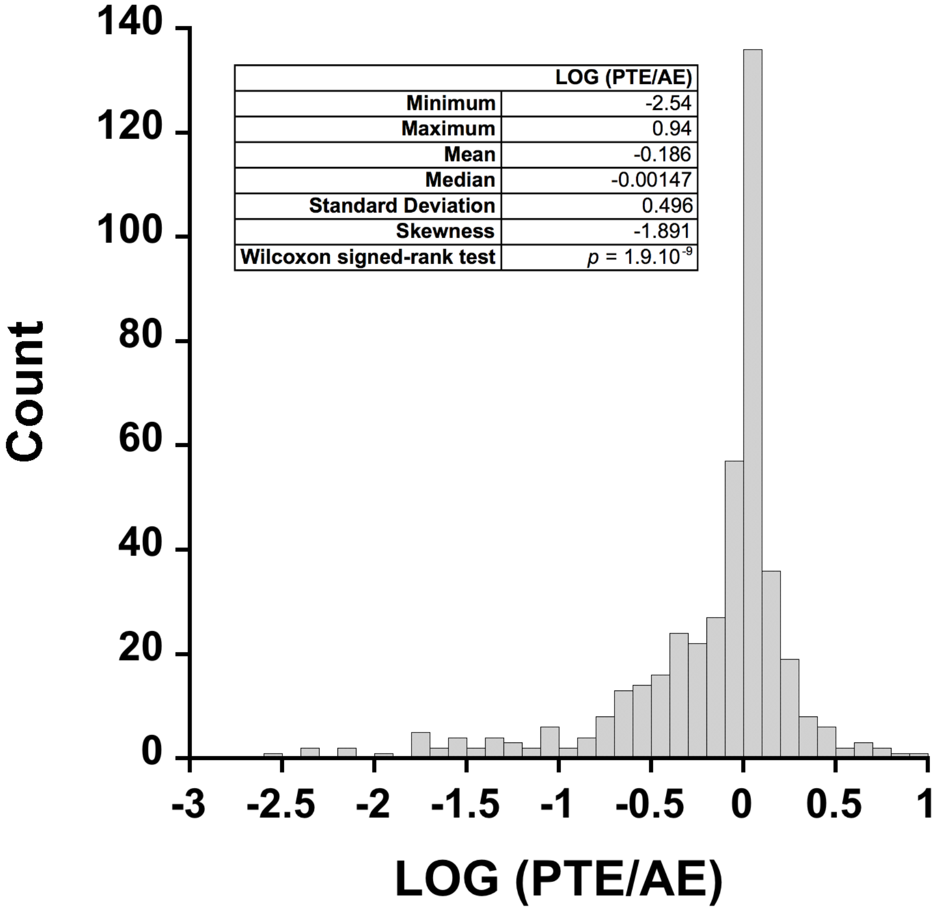

S1 Fig. Distribution of the difference of the log-transformed PTE and AE relative activities for each variant (n = 435). The distribution is significantly not centred on zero (Wilcoxon signed-rank test, $p < 0.0001$) and is skewed toward negative values.
