# Supplementary material for: Functional Trade-Offs in Promiscuous Enzymes Cannot Be Explained by Intrinsic Mutational Robustness of the Native Activity
Source: PLoS Genet. 2016 Oct 7;12(10):e1006305. doi: 10.1371/journal.pgen.1006305 (PMC5065130; doi:10.1371/journal.pgen.1006305)
Supplement: S2 Fig — (PDF) [file pgen.1006305.s012.pdf]

Functional trade-offs in promiscuous enzymes cannot be explained by intrinsic mutational robustness of the native activity

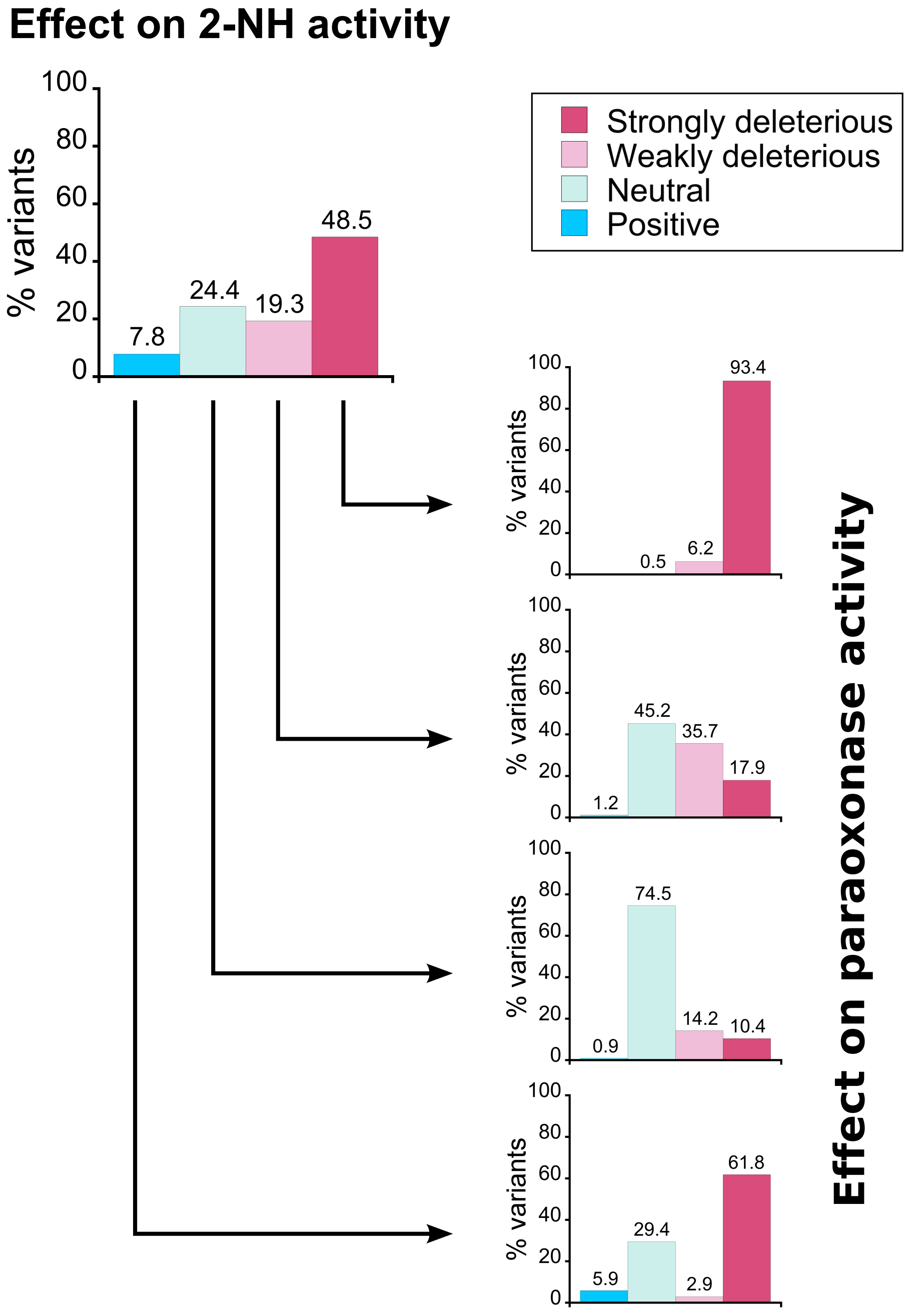

S2 Fig. Distribution of the effect of single trinucleotide substitutions on the native phosphotriesterase activity depending on their effect on the promiscuous arylesterase activity. Mutations are classified as strongly deleterious (>2-fold activity decrease relative to *wt*PTE), weakly deleterious (2-fold - 1.3-fold decrease), neutral (<1.3-fold change), and positive (>1.3-fold increase)**.**
