# Supplementary material for: Functional Trade-Offs in Promiscuous Enzymes Cannot Be Explained by Intrinsic Mutational Robustness of the Native Activity
Source: PLoS Genet. 2016 Oct 7;12(10):e1006305. doi: 10.1371/journal.pgen.1006305 (PMC5065130; doi:10.1371/journal.pgen.1006305)
Supplement: S1 Text — (PDF) [file pgen.1006305.s013.pdf]

Functional trade-offs in promiscuous enzymes cannot be explained by intrinsic mutational robustness of the native activity

Detailed Methods

Cloning of *wt*PTE into pID-Tet

The pID-Tet vector (Emond et al., in preparation) was specifically designed for the generation of variant libraries by transposon-based approaches [1] and its sequence does not contain recognition sequences for the following restriction enzymes (RE): MlyI (GAGTC(5/5), AcuI (CTGAAG(16/14), NotI (GC↓GGCCGC), EcoP15I (CAGCAG(25/27)) and FokI (GGATG(9/13)). A synthetic gene encoding *wt*PTE (lacking the RE recognition sequences listed above) was cloned in pID-Tet using NcoI and HindIII. The resulting pID-Tet-*wt*PTE plasmid was used for the construction of the TriNEx library of *wt*PTE. Gene synthesis was performed by GenScript (Piscataway, NJ, USA).

Preparation of TransDel and SubsNNN

DNA sequences corresponding to the TransDel transposon (containing the chloramphenicol resistance gene) and a Kanamycin resistance cassette (dubbed Insertion Replacement Cassette, IRC) were synthesized and cloned into pUC57 (GenScript, NJ, USA). Prior to in vitro transposition, TransDel was extracted from pUC57 by BglII digestion and recovered by gel electrophoresis and purification. Cassette SubsNNN was prepared from IRC by PCR using the following primers: 5'- [Phos] - ATGTC<u>GACTC</u>GACTAGTGCTTGGATT-CTCA - 3' and 5'- [Phos] - NNNGGGAT<u>GACTC</u>CATGGACTTCGC - 3' (MlyI sites underlined).

Generation of a substitution variant library from *wt*PTE using TriNEx

(1) Generation of transposon insertion library. Insertion of TransDel (a MuDel-like engineered transposon [1]) in pID-Tet plasmid containing the *wt*PTE gene was performed using *in vitro* transposition using 300 ng of plasmid, 50 ng of transposon and 0.22 µg MuA transposase (Thermo

Scientific) in 20 µL reaction volume. After incubation for 2 h at 30°C, the MuA transposase was heat-inactivated for 10 min at 75°C. DNA products were purified and concentrated in 7 µL deionized water using a DNA clean concentrator kit (Zymo Research). 2 µL of the purified DNA was used to transform *E. coli* Ecloni® 10G cells (Lucigen) by electroporation. The transformants (~50,000 CFUs) were selected on LB agar containing ampicillin (amp; 100 µg/mL) and chloramphenicol (cam; 34 µg/mL). The resulting colonies were pooled and their plasmid DNA extracted. Fragments corresponding to the *wt*PTE gene with the inserted transposon were obtained by NcoI/HindIII double restriction digestion. Upon gel extraction, they were ligated in pID-Tet. The ligation products were then transformed into electrocompetent *E. coli* Ecloni® 10G cells. Transformants selected on LB-amp-cam (~ 2.10^6 CFUs) were pooled and their plasmid DNA extracted, yielding TransDel insertion library of *wt*PTE.

(2) Generation of the library of trinucleotide substitution variants of *wt*PTE. TransDel insertion library plasmids were digested with MlyI to remove TransDel. Upon gel electrophoresis and recovery, the linearized pID-Tet-*wt*PTE plasmids were ligated with SubsNNN. After purification and concentration, these ligation products were transformed into electrocompetent *E. coli* Ecloni® 10G and the transformants (~ 2.10^6 CFUs) were selected on LB agar containing ampicillin (100 µg/mL) and kanamycin (50 µg/mL). Plasmids corresponding to the SubsNNN insertion library were extracted from the pooled bacterial colonies and subsequently digested with MlyI to remove the SubsNNN cassette. The linearized pID-Tet-*wt*PTE plasmids (with an random trinucleotide substitution) were recovered by gel electrophoresis, purified and self-circularized using T4 ligase (Thermo Scientific). The DNA was then transformated into electrocompetent *E. coli* Ecloni® 10G cells and transformants (~ 2.10^6 CFUs) were selected on LB-agar-amp. The resulting TriNex library was purified directly from the pooled bacterial colonies and stored in the form of a plasmid solution.

1. Baldwin AJ, Busse K, Simm AM, Jones DD. Expanded molecular diversity generation during directed evolution by trinucleotide exchange (TriNEx). Nucleic Acids Res. 2008;36(13):e77.
